# *Plasmodium knowlesi* Cytoadhesion Involves SICA Variant Proteins

**DOI:** 10.3389/fcimb.2022.888496

**Published:** 2022-06-23

**Authors:** Mariko S. Peterson, Chester J. Joyner, Stacey A. Lapp, Jessica A. Brady, Jennifer S. Wood, Monica Cabrera-Mora, Celia L. Saney, Luis L. Fonseca, Wayne T. Cheng, Jianlin Jiang, Stephanie R. Soderberg, Mustafa V. Nural, Allison Hankus, Deepa Machiah, Ebru Karpuzoglu, Jeremy D. DeBarry, Rabindra Tirouvanziam, Jessica C. Kissinger, Alberto Moreno, Sanjeev Gumber, Eberhard O. Voit, Juan B. Gutierrez, Regina Joice Cordy, Mary R. Galinski

**Affiliations:** ^1^ Emory National Primate Research Center, Emory University, Atlanta, GA, United States; ^2^ Emory Vaccine Center, Emory University, Atlanta, GA, United States; ^3^ Center for Tropical and Emerging Global Diseases, University of Georgia, Athens, GA, United States; ^4^ School of Chemical, Materials and Biomedical Engineering, University of Georgia, Athens, GA, United States; ^5^ Division of Animal Resources, Yerkes National Primate Research Center, Emory University, Atlanta, GA, United States; ^6^ The Wallace H. Coulter Department of Biomedical Engineering, Georgia Institute of Technology and Emory University, Atlanta, GA, United States; ^7^ Institute of Bioinformatics, University of Georgia, Athens, GA, United States; ^8^ Division of Pathology, Yerkes National Primate Research Center, Atlanta, GA, United States; ^9^ Department of Pediatrics, Emory University School of Medicine, Atlanta, GA, United States; ^10^ Department of Genetics, University of Georgia, Athens, GA, United States; ^11^ Division of Infectious Diseases, Department of Medicine, Emory University School of Medicine, Atlanta, GA, United States; ^12^ Department of Pathology and Laboratory Medicine, Emory School of Medicine, Atlanta, GA, United States; ^13^ Department of Mathematics, University of Georgia, Athens, GA, United States

**Keywords:** malaria, antigenic variation, rhesus monkey (*Macaca mulatta*), gastritis, anemia, infected erythrocytes, *Plasmodium falciparum*, histopathology (HPE)

## Abstract

*Plasmodium knowlesi* poses a health threat throughout Southeast Asian communities and currently causes most cases of malaria in Malaysia. This zoonotic parasite species has been studied in *Macaca mulatta* (rhesus monkeys) as a model for severe malarial infections, chronicity, and antigenic variation. The phenomenon of *Plasmodium* antigenic variation was first recognized during rhesus monkey infections. *Plasmodium*-encoded variant proteins were first discovered in this species and found to be expressed at the surface of infected erythrocytes, and then named the Schizont-Infected Cell Agglutination (SICA) antigens. SICA expression was shown to be spleen dependent, as SICA expression is lost after *P. knowlesi* is passaged in splenectomized rhesus. Here we present data from longitudinal *P. knowlesi* infections in rhesus with the most comprehensive analysis to date of clinical parameters and infected red blood cell sequestration in the vasculature of tissues from 22 organs. Based on the histopathological analysis of 22 tissue types from 11 rhesus monkeys, we show a comparative distribution of parasitized erythrocytes and the degree of margination of the infected erythrocytes with the endothelium. Interestingly, there was a significantly higher burden of parasites in the gastrointestinal tissues, and extensive margination of the parasites along the endothelium, which may help explain gastrointestinal symptoms frequently reported by patients with *P. knowlesi* malarial infections. Moreover, this margination was not observed in splenectomized rhesus that were infected with parasites not expressing the SICA proteins. This work provides data that directly supports the view that a subpopulation of *P. knowlesi* parasites cytoadheres and sequesters, likely *via* SICA variant antigens acting as ligands. This process is akin to the cytoadhesive function of the related variant antigen proteins, namely Erythrocyte Membrane Protein-1, expressed by *Plasmodium falciparum*.

## Introduction

Malaria is a blood-borne disease of major global importance that is caused by infection with parasitic protozoa from the genus *Plasmodium* and afflicts hundreds of millions of people annually (World Health Organization, 2021). Malarial illness presents with a wide array of clinical signs and symptoms that range from minimal to severe and life-threatening (reviewed in Miller et al., 2013; Milner, 2018). Parasite species and genotype, variant antigen expression, infected red blood cell (iRBC) cytoadhesion and sequestration in the microvasculature, iRBC concealment, and divergent host genetics and host immune responses contribute to the range of disease severity observed during *Plasmodium* infections (Miller et al., 2002; Baird, 2013; Miller et al., 2013; Wassmer et al., 2015; Fonseca et al., 2017). Given the complexities of malaria, the continued rise in drug resistance, and the general need for new parasite and possible host-directed antimalarial treatments, new insights into host-parasite interactions and mechanisms that govern malarial disease progression and resilience are of great importance (Miller et al., 2013; Ashley et al., 2014; Amato et al., 2018).

*Plasmodium knowlesi* is a zoonotic simian malaria parasite that causes a spectrum of malarial disease manifestations in humans in Southeast Asia (Singh et al., 2004; Cox-Singh et al., 2008; Cox-Singh and Singh, 2008; Daneshvar et al., 2009; William et al., 2011; Barber et al., 2017; Barber et al., 2018; Rajahram et al., 2019). This parasite naturally infects kra monkeys (*Macaca fascicularis*) and other macaque species that are native to Southeast Asia, and these animals control their parasitemia without treatment, leading to chronic infections (Knowles and Gupta, 1932; Garnham, 1966; Collins et al., 1992). In contrast, if left untreated with antimalarial drugs*, P. knowlesi* causes severe, lethal disease nearly universally in Indian rhesus macaques (*Macaca mulatta*) (Knowles and Gupta, 1932; Garnham, 1966; Coatney et al., 1971; Spangler et al., 1978; Coatney et al., 2003). Relevantly, key differences between the transcriptomic responses of *M. fascicularis* and *M. mulatta* to *P. knowlesi* infection were recently documented by Gupta and colleagues, bringing emphasis to the utility of cross-species comparisons (Gupta et al., 2021; Gupta et al., 2022).

Severe *P. knowlesi* infections in humans have been specifically associated with gastrointestinal (GI) pathology (Cox-Singh et al., 2008; Barber et al., 2013). Autopsy studies from fatal *P. knowlesi* human cases to better understand this phenomenon and other disease factors are scarce due to cultural considerations (Cox-Singh et al., 2010). Autopsy reports from *P. falciparum* cases are more common (Menezes et al., 2012), and *P. falciparum* trophozoite and schizont (T/S)-iRBCs have been documented in the microvasculature of the GI tract, with some studies demonstrating associated histopathology and GI symptoms (Romero et al., 1993; Seydel et al., 2006). Studies with rhesus macaques have reported *P. knowlesi* iRBCs in the microvasculature of the GI tract and other tissues, suggestive of cytoadhesion and sequestration, despite – unlike *P. falciparum* – the peripheral circulation of all asexual blood-stage developmental forms from the life cycle of this species (Miller et al., 1971b; Spangler et al., 1978). However, potential associations between the *P. knowlesi* parasite burden in the microvasculature of various tissues, adhesive interactions, and tissue damage have yet to be explored.

While all *P. knowlesi* blood-stage forms circulate, a subpopulation of *P. knowlesi* iRBCs may cytoadhere and sequester in tissues due to receptor-ligand interactions mediated by schizont-infected cell agglutination (SICA) proteins, which are variant antigens expressed by *P. knowlesi* that are related to and share features with *P. falciparum* erythrocyte membrane protein 1 (*Pf*EMP1) and the genes that encode them (Korir and Galinski, 2006; Bunnik et al., 2019). SICA and *Pf*EMP1 are large multi-domain proteins (~200-300 kDa) (Howard et al., 1983; Leech et al., 1984) that are encoded by large, diverse multigene families termed *SICAvar* and *var*, respectively (Baruch et al., 1995; Smith et al., 1995; Su et al., 1995; Al-Khedery et al., 1999). The expressed SICA proteins become inserted in the iRBC membrane with variant domains exposed at the surface of SICA positive (SICA[+]) iRBCs (Howard et al., 1983), and their expression in rhesus monkeys has been associated with virulence (Barnwell et al., 1983b). *SICAvar* gene and protein expression are dependent upon the presence of the spleen, with SICA[+] parasites taking on a SICA negative (SICA[-]) phenotype after passage in splenectomized rhesus (Barnwell et al., 1982; Barnwell et al., 1983a). The SICA[-] phenotype is characterized by downregulated *SICAvar* and SICA protein expression, notably to an ‘off state’ (Lapp et al., 2013). Infection of spleen-intact rhesus monkeys with SICA[-] parasites may result in milder disease, or return of SICA expression along with severe outcomes and fatality (Barnwell et al., 1983a). Similarly in *in vitro* cultures, *SICAvar* gene family expression was shown to be downregulated (Lapp et al., 2015). *Plasmodium falciparum* variant antigen expression has also been shown to be spleen-dependent *in vivo* in nonhuman primate (NHP) models and human infections (David et al., 1983; Hommel et al., 1983; Bachmann et al., 2009). SICA protein expression may mediate cytoadhesion and sequestration of a subpopulation of *P. knowlesi* iRBCs in the microvasculature of tissues, akin to *P. falciparum* T/S-iRBCs populations that express *Pf*EMP-1 (Aley et al., 1984; Leech et al., 1984; Biggs et al., 1991; Biggs et al., 1992), enabling widespread host-parasite receptor-ligand interactions and sequestration of *P. falciparum* iRBCs in the tissues (reviewed in Smith, 2014). Whether and to what degree *P. knowlesi* parasite sequestration concomitant with local and systemic inflammation may associate with enhanced symptomology and histopathology, which is evident with *Pf*EMP-1 expression, cytoadhesion, and sequestration (reviewed in Craig et al., 2012; Smith et al., 2013; Wahlgren et al., 2017; Lee et al., 2019), are questions of prime interest.

The experimental *P. knowlesi*-rhesus macaque infection model has provided significant insights into malarial immunity, notably, the major discovery of malarial antigenic variation during the course of longitudinal infections, which entails changes in the antigenic phenotype of iRBCs in concert with the development of antigen-specific immunity (Brown and Brown, 1965). This model then enabled the first identification and characterization of *Plasmodium*-encoded iRBC surface-exposed immunodominant variant proteins, namely the *P. knowlesi* SICA proteins (Barnwell et al., 1983a; Howard et al., 1983; Howard and Barnwell, 1984a; Howard and Barnwell, 1984b; Howard and Barnwell, 1985), which preceded the identification of *Pf*EMP1 based on similar extraction and characterization procedures (Howard et al., 1984; Leech et al., 1984; Howard et al., 1988).

The longitudinal *P. knowlesi* sporozoite-induced infections in malaria-naïve rhesus monkeys presented here delve into histopathology questions pertinent to understanding *P. knowlesi* infections in humans. These experiments were generated by the Malaria Host-Parasite Interaction Center (MaHPIC) systems biology research consortium, which previously reported the utilization and integration of multi-omic technologies to investigate and compare host-pathogen interactions during acute, chronic, and relapse phases of malarial infections caused by *Plasmodium cynomolgi* or *Plasmodium coatneyi* (Joyner et al., 2016; Joyner et al., 2017; Tang et al., 2017; Cordy et al., 2019; Joyner et al., 2019; Tang et al., 2019; Debarry et al., 2020), and *P. knowlesi* in kra and rhesus monkeys (Gupta et al., 2021; Peterson et al., 2021; Gupta et al., 2022). Within the rhesus monkey cohorts presented here, longitudinal clinical data and pathological relationships were documented and modest relationships were identified between organ pathology and parasite tissue load assessed in 22 tissue types. Of note, extensive margination of SICA[+] iRBCs was observed along the vascular endothelium of the GI tract. Strikingly, SICA[+] iRBC margination was observed with abundant juxtaposition of iRBCs with the endothelium, but this was not observed in the tissues of splenectomized rhesus infected with SICA[-] parasites. In summary, the rhesus macaque animal model was utilized to systematically characterize clinical features of malarial disease, *P. knowlesi* sequestration and tissue pathology, and the results point to SICA proteins functioning at the interface of iRBCs and the GI tract.

## Materials and Methods

### Animal Use

All NHP experiments were performed at the Yerkes National Primate Research Center (YNPRC), an AAALAC Internationally accredited facility. All experimental, surgical, and necropsy procedures were approved by Emory’s Institutional Animal Care and Use Committee (approval number: PROTO201700484-YER-2003344-ENTRPR-A) and the Animal Care and Use Review Office of the US Department of Defense and followed accordingly. All animal procedures used are consistent with the ARRIVE guidelines (Percie Du Sert et al., 2020).

Sixteen rhesus monkeys were assigned within six cohorts (**Supplemental Table 1**; Experiments 30, 06, 33, 34, 35 and Spx; splenectomy experiment). Eleven animals within four of these cohorts were designated for sequential iterative longitudinal *P. knowlesi* sporozoite-induced infection experiments (**Supplemental Figures 1**–**4**), aiming to understand the virulence of *P. knowlesi* blood-stage infections in rhesus monkeys. Three animals were designated as control animals and two for a final SICA[-] blood-stage infection experiment requiring splenectomy. The study involved only male monkeys to avoid confounding blood loss and anemia due to menstruation. All rhesus were of Indian origin and all but two were born and raised at the YNPRC; the animals with codes 13_116 and 13_136 were acquired from a domestic breeding facility and quarantined per YNPRC standard operating procedures before being involved in this study. All animals were healthy and housed socially, with 12 h light-dark cycles, and received environmental enrichment with food, foraging activities, and other physical manipulanda, compliant with the Animal Welfare Act and the Guide for the Care and Use of Laboratory Animals. The animals received training prior to the initiation of the experiments to familiarize them with daily or twice-daily ear stick collections of small blood volumes as described previously with kra monkey infections (Peterson et al., 2021). End points for each cohort were pre-determined to understand the pathophysiology of infection longitudinally. All animals were necropsied at the pre-determined end points by anesthetizing them using ketamine and then euthanizing *via* intravenous administrations of barbiturates, an acceptable method of euthanasia for NHP per recommendations of the “AVMA Guidelines for the Euthanasia of Animals”.

### Parasite Isolates and Inoculations

Eleven malaria naïve rhesus (cohorts E30, E06, E33 and E35) were infected intravenously with cryopreserved *P. knowlesi* clone Pk1(A+) sporozoites derived from the Malayan Strain of *P. knowlesi* (Barnwell et al., 1983a; Lapp et al. 2018) (kindly provided by John W. Barnwell, Centers for Disease Control and Prevention, Atlanta, GA). In addition, two previously infected and then splenectomized adult male rhesus were infected intravenously with cryopreserved *P. knowlesi* clone Pk1(A-)1- iRBCs, a cloned parasite line that is phenotypically negative for expression of the *SICAvar* family and encoded SICA proteins, derived from the passage of Pk1(A+) cloned parasites through a splenectomized monkey (Barnwell et al., 1983a).

### Subcurative Drug Treatments

To prevent hyperparasitemia and mortality of rhesus monkeys that were not sacrificed while parasitemia was escalating, subcurative antimalarial treatments were administered when the peripheral parasitemia reached approximately 1% of infected RBCs and the parasite replication rate was not decreasing. Subcurative treatments consisted of artemether at 1 mg/kg (for cohorts E30 and E35) or chloroquine at 5 mg/kg (for cohort E33), administered intramuscularly. Further descriptions and experimental details by cohort are discussed in **Supplemental Table 1**.

### Sample Collections

Standard ear-stick procedures were performed to obtain capillary blood samples in EDTA for complete blood counts (CBCs) and monitoring of parasitemias. Larger volume blood collections were performed from the saphenous or femoral vein into EDTA under ketamine anesthesia.

#### Telemetry

The animals in cohorts E30 and E06 had telemetry devices surgically implanted prior to infection for real-time monitoring of temperature and other vital signs. Raw temperature data processing, definition of normal temperature range, and determination of febrile threshold and time-to-temperature responses were conducted as described previously (Gatton and Cheng, 2002; Peterson et al., 2021 and Brady et al, manuscript in preparation).

### Calculation of Reticulocyte Production Index

RPI calculation was performed to correct for raw reticulocyte count in the setting of anemia, and determine appropriateness of bone marrow response, as described previously (Peterson et al., 2021).

#### Quantification of Serum Erythropoietin

Serum erythropoietin for samples collected at baseline and days 8-10 post-inoculation was measured using the Human Erythropoietin Quantikine IVD ELISA kit (R&D Systems) on undiluted serum as described previously (Peterson et al., 2021).

#### Multiplex Cytokine Array

Plasma samples collected at baseline and between days 8-10 post-inoculation with sporozoites were used to quantify the concentration of 29 cytokines using the Cytokine 29-Plex Monkey Panel (Thermofisher, Inc) according to the manufacturer’s suggested protocol (serum sample dilution of 1:2). Per the manufacturer, this kit was developed and validated for rhesus monkeys. Data were analyzed using the Luminex software suite. A floor of the minimum detected value for each analyte was then substituted for undetectable values, and then the data were log_10_ transformed before statistical analyses. Analytes included in this kit were EGF, Eotaxin, FGF, G-CSF, CM-CSF, HGF, IFNγ, IL-10, IL-12, IL 15, IL 17, IL-1b, IL-1RA, IL-2, IL-4, IL-5, IL-6, IL-8, IP-10, I-TAC, MCP-1, MDC, MIF, MIG, MIP-1α, MIP-1β, Rantes, TNFα, and VEGF. Only cytokines with statistically significant changes from baseline are discussed.

### Parasite Enumeration

Blood smears were monitored for the presence of parasites once daily between 1 PM and 3 PM until the infections became patent, after which, parasitemia was monitored twice daily (8 AM, schizonts; and 1-3 PM ring stages). Smear preparation, examination, and enumeration were performed as described previously (Peterson et al., 2021). Cumulative parasitemia was calculated for each animal by adding together the daily parasitemia (parasites/µl) from the day of inoculation to the day of necropsy.

### Quantification of Parasite Replication Rate

The replication rate of parasites was calculated as described previously (Fonseca et al., 2017; Peterson et al., 2021). Only the first peak was considered because it had the most robust sample size.

#### Tissue Collection, Preservation, and Pathology Analysis

Twenty-two tissue sample types were collected at necropsy from all infection experiments (**Figure 5**), except cohort E33 which was limited to the tissues of most interest, namely liver, lung, kidney, spleen, adrenal gland, bone marrow (BM), stomach, duodenum, jejunum, and colon. The tissues were fixed processed, sectioned, and stained with hematoxylin and eosin (H&E) as described previously (Peterson et al., 2021). The slides were examined using regular bright field light microscopy and also under polarized light to highlight hemozoin crystals, which are birefringent, distinguishing them from hemosiderin (Lawrence and Olson, 1986). Sections for transmission electron microscopy (TEM) (in 2.5% glutaraldehyde) were also collected, stored, and analyzed using appropriate equipment and technologies (Sheehan and Hrapchak, 1980). All tissue slides were blinded and randomized for diagnosis and characterization of histopathology. Tissue scoring was performed as previously described (Peterson et al., 2019; Peterson et al., 2021).

### Infected RBC Quantification Within Tissues

iRBC densities in the tissues were determined by counting the number of iRBCs in ten high power fields (HPFs) (1000x) under oil immersion on a standard light microscope (Milner et al., 2013; Joice et al., 2014; Milner et al., 2015; Peterson et al., 2019). All sections were randomized and blinded. Standardized tissue densities, indicating relative differences between acute and chronic infection, taking into account parasitemia, were determined by dividing the parasite tissue density of each monkey in each tissue by the sum of all the parasite tissue densities in each group (acute and chronic infection). The resultant proportions were combined in a vector and binned to create a color scale. The color scale for a parasite distribution heat map was created using the bin_data command in the mltools package in R, with a bin value of seven, and a quantile bin type. The colors in the heat map are from the Blues Brewer Color Palette from the RColorBrewer package in R (Holtz, 2018), imported into Adobe Illustrator as.ase files.

### Infected RBC Margination

For quantification of marginated iRBCs, 20 parasitized blood vessels were quantified, or 350 total vessels, whichever was observed first. Fields were examined at 1000x under oil emersion, on a standard light microscope. Infected RBCs appearing to touch the endothelium were scored as marginated, others in the lumen were classified as non-marginated. The proportion of marginating parasites in each organ was calculated as the number of marginated iRBCs divided by the total number of iRBCs. Analysis was performed on tissues from infected rhesus and splenectomized rhesus.

### Transmission Electron Microscopy

TEM was performed on infected duodenum tissue from selected monkeys. Tissue grids were prepared by primary fixation in 2.5% glutaraldehyde, followed by washing, secondary fixation in 1% osmium tetroxide, dehydration in graded ethanol solutions and staining with uranyl acetate, treatment with 100% propylene oxide, infiltration of resin, and resin embedding (Sheehan and Hrapchak, 1980). Semi-thin tissue sections were stained with toluidine blue and examined by light microscopy prior to grid preparation to aid in the proper orientation of samples. Ultrathin grid sections were prepared at 10-20 nm thickness and examined by electron microscopy (JEOL model 1011), and image acquisition (Gatan model 785).

### Computation of the Removal of Uninfected RBCs

The degree of removal of uninfected RBCs was computed with a recursive mathematical model of RBC dynamics as previously performed for macaques infected with *P. cynomolgi* (Fonseca et al., 2017).

#### Statistical Analyses

**Figure 1:**
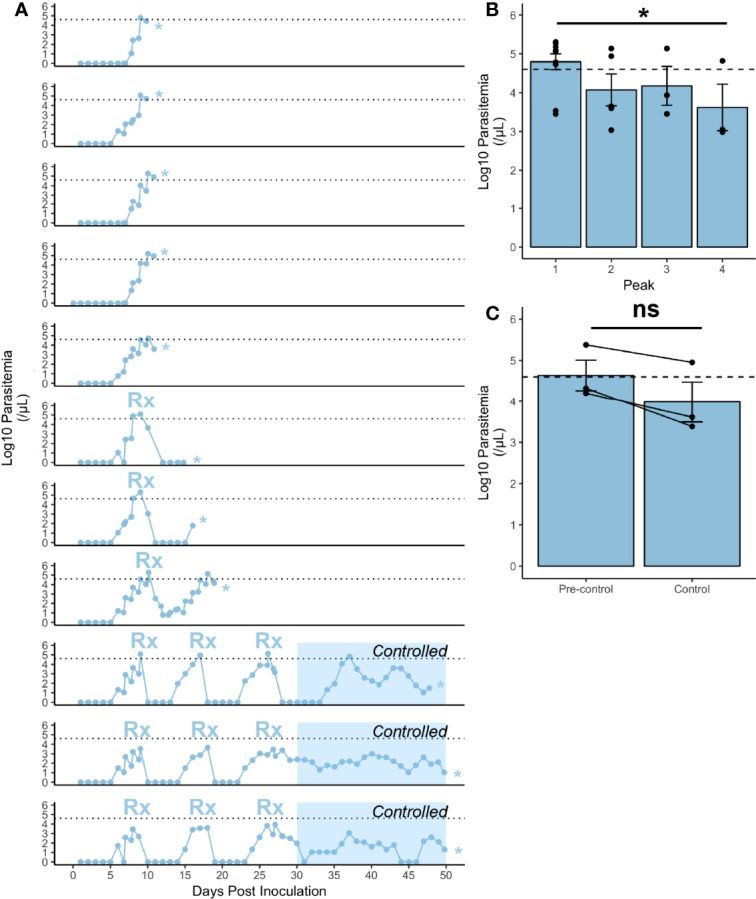
Infection characteristics of rhesus monkeys infected with *P. knowlesi.* **(A)** Log_10_-transformed parasitemia curves for 11 monkeys infected with *P. knowlesi* sporozoites. Treatment threshold (1% parasitemia = 40,000 parasites/μL) is indicated by the dotted line, and necropsy day is marked with an asterisk. Time points of subcurative treatment with either artemether or chloroquine are indicated by the “Rx” symbol. The interval in which the monkeys controlled parasitemia without the need for subcurative treatment is indicated by blue shading labeled “Controlled”. **(B)** Average log_10_-transformed parasitemias for the primary and recrudescent peaks showing gradual reduction in magnitude. The primary parasitemic peak was compared to the final recrudescence and found to be statistically significantly different *via* paired t-test comparison (*p-value < 0.05). **(C)** Cumulative parasitemia prior to, and after parasitemic control (30 dpi) shows similar parasitemia burdens between earlier and later times in infection *via* paired t-test comparison. ns (not significant) =p−value > 0.05. Error bars represent standard error of the mean.

Data were generally divided into two categories for these analyses. Samples and data acquired during the timeframe in which the monkeys required subcurative treatment were considered “pre-control,” and those thereafter were considered “controlled.” (**Figure 1** and **Supplemental Figures 1**–**4**). Statistics and figures were produced in R Studio, under R version 3.4.3 (R-Core-Team, 2021) GUI version 1.70. Pairwise comparisons were performed using Welch’s *t*-test and paired t-tests, and adjusted using the false discovery rate method, where appropriate. Multiple comparisons were performed using ANOVA analysis, including repeated measures ANOVA, and the Tukey’s HSD post-hoc comparison tests. Repeated measures ANOVA and associated post-hoc comparisons were performed using the psycho package v. 0.4.9 in R (Makowski, 2019). Associations were tested using the Pearson’s correlation test, and hierarchical multiple linear regression analyses. Multicollinearity was assessed using the olsrr package v. 0.5.2. in R (Aravind, 2018). Graphs were prepared using the ggplot2 package v. 3.1.1 (Wickham et al., 2019) and the Brewer Color Palette in RStudio (Holtz, 2018). Comparisons were considered significant with raw or adjusted *p*-values below 0.05.

## Results

The experimental cohorts from this study (E06, E33, E34, and E35), including the number of animals, sporozoite inoculations, monitoring, treatments, etc., are detailed in **Supplemental Table 1**. Schematics with parasitemias and sampling points for each animal by cohort are included in **Supplemental Figures 1**–**4**.

### Rhesus Monkeys Control Acute Blood-Stage Infections With Sub-Curative Therapeutic Intervention

Rhesus monkeys (n=11) inoculated with *P. knowlesi* sporozoites became patent 6-8 dpi and showed escalating parasitemia 7-10 dpi (**Figure 1A** and **Supplemental Figures 1**–**4**). The parasite replication rate in the rhesus monkeys was 23.36-fold ± 2.91 (mean ± SEM), and showed no signs of deceleration, supporting the notion that these animals were not controlling their infections (**Figure 1A**) (Fonseca et al., 2017). As expected from earlier studies (Brown and Brown, 1965), subcurative drug treatments led to the establishment of chronic infections (**Figure 1A**, bottom 3 panels). Per the study design used here, a subcurative dose of artemether (**Supplemental Figures 1****, 4**) or chloroquine (**Supplemental Figure 3**) was administered intramuscularly when the parasitemia reached greater than or equal to 1% with no evidence of control (**Figure 1A**). This pharmacological regimen differs from the originally reported use of quinine (Brown and Brown, 1965). The drug dampened the parasitemias to sub-patent levels, which then recrudesced, with incremental reduction of parasitemia spikes to below the 1% parasitemia treatment threshold until the rhesus no longer required subcurative treatment and the infection became chronic (**Figures 1A, B**). The recrudescent and cumulative parasitemias were calculated and compared longitudinally (**Figures 1B, C**). Paired t-test analysis of the final recrudescent peak prior to necropsy to the primary peak was statistically significantly lower (p-value = 0.04), consistent with the establishment of a controlled infection without antimalarial treatment (**Figure 1B**). The cumulative parasitemia prior to and after control was not statistically significant (**Figure 1C**). Altogether, these experiments reliably produced *P. knowlesi* parasitemia kinetics in rhesus monkeys, as expected from previous studies (Knowles and Gupta, 1932; Garnham, 1966; Coatney et al., 1971; Collins et al., 1992; Coatney et al., 2003).

**Figure 2:**
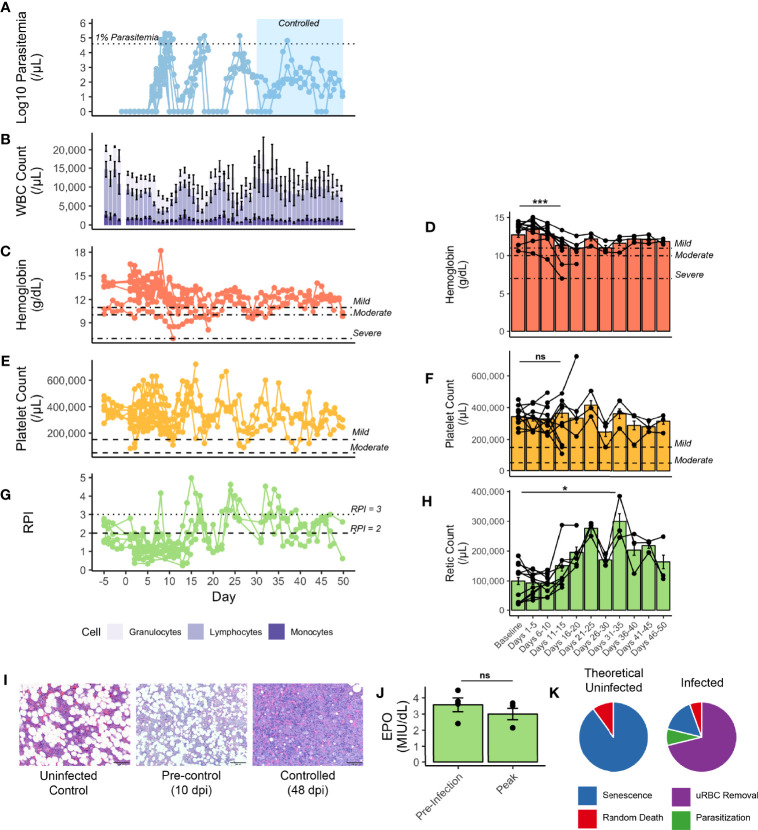
Hematological parameters of rhesus monkeys infected with *P. knowlesi*. **(A)** Longitudinal faceted plots showing parasitemia, with treatment threshold and control interval indicated, compared with **(B)** white blood cell count with population fractions indicated, **(C)** hemoglobin with mild, moderate, and severe anemia thresholds labeled (at 11 g/dL, 10 g/dL, and 7 g/dL, respectively), with **(D)** five day hemoglobin average, **(E)** platelet count with mild and moderate thrombocytopenia labeled (at 150,000 platelets/mL and 75,000 platelets/μL, respectively), with **(F)** five day platelet average, **(G)** reticulocyte production index (with RPI of 2 and of 3 labeled), and **(H)** five-day average of reticulocyte counts. **(I)** 600x H&E-stained tissue sections of bone marrow from an uninfected control monkey, a monkey prior to controlling parasitemia, and a monkey after controlling parasitemia. **(J)** Erythopoietin levels from select monkeys demonstrating no difference between baseline and 10 dpi, prior to antimalarial treatment. **(K)** Mathematical modeling predicting the relative proportions of infected and uninfected red blood cell removal. Statistical comparisons were performed using paired t-tests. ***p-value < 0.0005, *p-value < 0.05, and ns (not significant) = p-value > 0.05. Error bars represent standard error of the mean.

Rhesus monkey host responses were examined during the acute phase of infection, when they were unable to naturally control their infections, and compared after administration of sub-curative antimalarial therapy. Hematological parameters were evaluated to study the temporal development and degree of severity of anemia and thrombocytopenia. By 10-11 dpi, all rhesus had high escalating parasitemias requiring subcurative treatment (**Figures 1A**, **2A**). White blood cells and their subset populations rose corresponding with troughs in parasitemia (**Figure 2B**). While one animal experienced a hemoglobin nadir as low as 7 g/dL, the hemoglobin levels subsequently stabilized post-treatment at nadirs of 10.11 g/dL ± 0.45, an average of 75.45% ± 2.59% relative to baseline (**Figure 2C**). 5-day averages were calculated to compare hematological parameters longitudinally, and the hemoglobin nadirs, which occurred 11-15 dpi were statistically significantly lower than baseline (p-value = 1.14x10^-4^) (**Figure 2D**). Interestingly, the animals did not experience dramatic platelet nadirs, the lowest being 75,000 platelets/uL (mean 163,909 ± 19,636.91) (**Figure 2E**). Platelet levels at 11-15 dpi were not different from baseline (**Figure 2F**). Platelet nadirs corresponded with rises in parasitemia (**Figures 2A, E**).

Insufficient RBC production to match RBC removal contributes to and exacerbates the development of anemia in rhesus monkeys infected with either *P. cynomolgi* or *P. coatneyi* (Moreno et al., 2013; Joyner et al., 2016; Tang et al., 2017; Cordy et al., 2019; Joyner et al., 2019). Here it was hypothesized that insufficient erythropoiesis could similarly contribute to the development of anemia during the acute stage of infections with *P. knowlesi*, prior to administering subcurative treatment regimens. As such, several metrics for BM function were examined. Indeed, prior to the administration of subcurative treatment, the Reticulocyte Production Index (RPI) remained below 2, the threshold that indicates an appropriate BM response to a drop in hemoglobin. However, it increased after the administration of subcurative treatment in pulses corresponding to parasitemia (**Figures 2A, G**). The peak output of reticulocytes, as measured by peripheral reticulocyte count occurred at 31-35 dpi, which was significantly higher than baseline counts (p-value = 0.03) and occurred around the same time as parasitemic control (**Figures 2A, H**). Histologically, BM “at rest” normally contains adipose tissue as a proportion of age, along with platelet and red and white blood cell progenitors. Microscopic examination of BM samples revealed that BM expansion was stunted in the rhesus monkeys prior to treatment as is evidenced by a similar proportion of fat droplets seen between baseline prior to infection and a representative sample taken at 10 dpi (**Figure 2I**). With activation, myeloid and erythroid precursors expand and crowd out fat droplets, as can be shown in a representative section taken at 48 dpi, suggesting eventual BM recovery later during the infection (**Figure 2I**). Serum erythropoietin was measured in a subset of monkeys prior to treatment and found to not be significantly different from baseline, consistent with a delayed BM response to infection-induced anemia (**Figure 2J**).

Since the drop in hemoglobin, though relatively mild, could not be explained by parasite-mediated destruction alone, the magnitude of destruction of uninfected RBCs was predicted using a mathematical model (Fonseca et al., 2016; Fonseca et al., 2017; Peterson et al., 2021). Analysis of a rhesus monkey from cohort E35 (**Supplemental Table 1**), demonstrated that destruction of uninfected RBCs accounted for 71% of all RBCs removed during the experiment, while only 8% were lost due to parasitization (**Supplemental Figure 5** and **Figure 2K**), and a compensatory release of reticulocytes with each subcurative treatment (**Figures 2A, G**).

**Figure 3:**
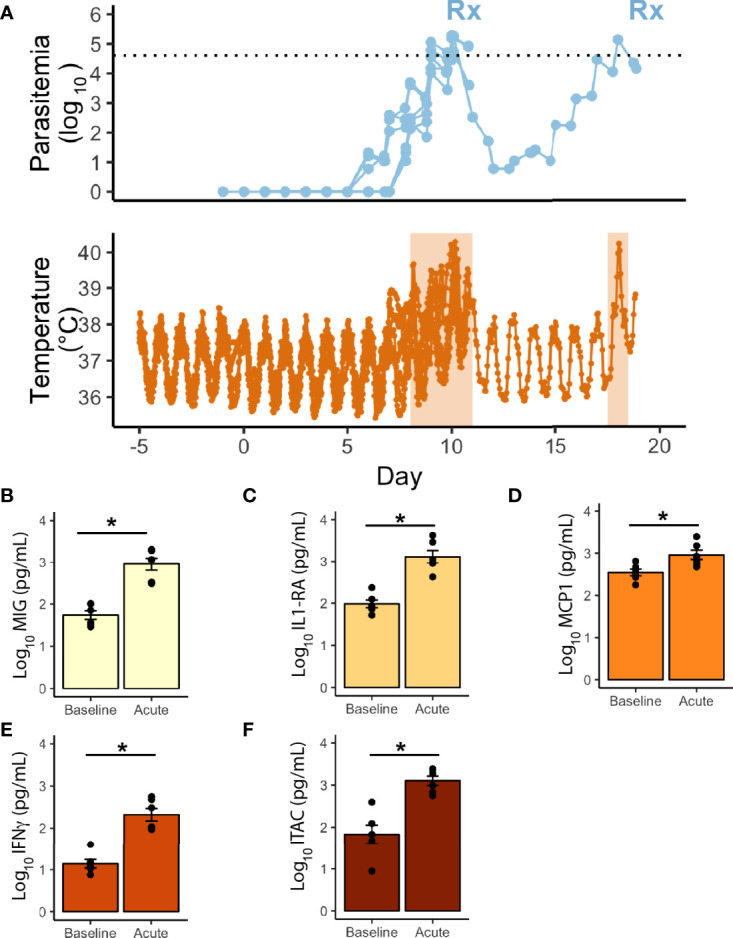
Telemetric temperature response to *P. knowlesi* parasitemia. **(A)** Top: Longitudinal parasitemia curve of the 6 monkeys with implanted telemetry devices, with treatment threshold indicated by dotted line. Bottom: Temperature data obtained from implanted telemetry devices indicating rises in temperature above each individual’s baseline corresponding to rises in parasitemia. Febrile periods are marked with orange shading. Statistically significant cytokine results from a 29-cytokine array including **(B)** MIG, **(C)** IL1-RA, **(D)** MCP1, **(E)** IFNγ, **(F)** ITAC. Statistical comparisons were performed using paired t-tests, with FDR correction. *Corrected p-value<0.05. Error bars represent standard error of the mean.

Core body temperature was studied as a proxy for inflammation. Telemetry devices were surgically implanted before experimental infection into two cohorts of monkeys (E06 and E30) to collect densely sampled and real-time temperature data (6 rhesus; **Figure 3A**, and **Supplemental Table 1**). The parasitemia at which the monkeys waged a temperature response (pyrogenic threshold) was determined by comparing each monkey’s baseline to the nearest parasitemia corresponding to when the temperature rose above the individual’s baseline. The rhesus monkeys responded on average to a parasite density of 364/μl during the acute infection period and took approximately 8 days post-infection to do so, including one animal that waged a fever at early recrudescent peaks, and at a higher threshold than its primary peak (390/μl vs 29,070/μl). A 29-cytokine array was also performed to compare inflammatory cytokines at baseline with peak parasitemia, occurring in the febrile period. Of these, five were statistically significantly elevated, including IL1RA and IFNγ, monocyte associated markers MIG and MCP1, and T-cell associated factor, ITAC (**Figures 3B–F**).

### Major Histopathological Findings in the Liver, Lungs, Kidney, and Stomach

Although uncontrolled parasitemia is a primary contributor to severe disease in rhesus monkeys, prior studies with rhesus have not reported comparative evaluations of histopathological changes that occur during *P. knowlesi* infections, particularly when overwhelming parasitemia is being limited by antimalarial treatments (Knowles and Gupta, 1932; Knisely et al., 1964; Garnham, 1966). Therefore, this study design included an extensive analysis of H&E-stained tissue sections from 22 different organs from infected monkeys at the time of sacrifice.

**Figure 4:**
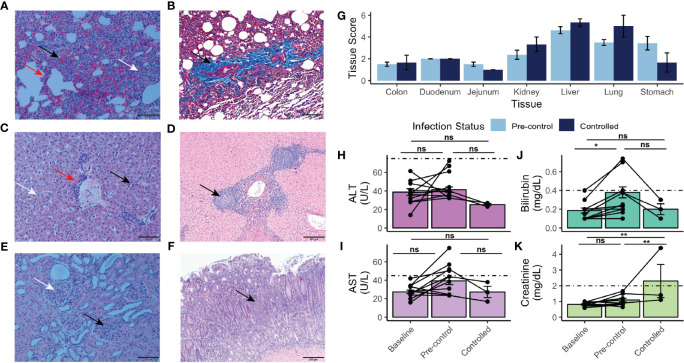
Histopathology, tissue damage, and results of chemistry analysis of rhesus monkeys infected with *P. knowlesi*. **(A)** H&E-stained section of rhesus lung under polarized light microscopy (scale bar = 100 μm). Hemorrhage (black arrow), interstitial thickening and infiltration (red arrow), and diffuse hemozoin (white arrow) are indicated. **(B)** Masson’s trichrome-stained section (scale bar = 200 μm) of rhesus lung highlighting fibrotic changes with infection (black arrow, and bright blue color of fibrin). **(C)** H&E-stained section (scale bar = 100 μm) of rhesus liver showing periportal inflammation (red arrow), sinusoidal congestion (black arrow), and parenchymal hemozoin deposition (white arrow). **(D)** H&E-stained section (scale bar = 200 μm) of liver from a monkey infected for 48 days showing unique lobular mononuclear infiltration in the periportal area (black arrow). **(E)** H&E-stained section (scale bar = 100 μm) of kidney under polarized light showing hypercellular glomeruli (black arrow) and abundant hemozoin (white arrow). **(F)** H&E stained section (scale bar = 200 μm) of stomach demonstrating mononuclear infiltration of the mucosa (black arrow), and submucosal edema, consistent with gastritis. **(G)** Semi-quantitative tissue scores of infected monkeys necropsied prior to and after parasitemic control. No statistically significant differences were noted using Tukey’s HSD post-hoc analysis. **(H)** ALT and **(I)** AST measurements taken at baseline, pre-control, and after control indicating no statistically significant difference relative to baseline. **(J)** Bilirubin and **(K)** creatinine measurements taken at baseline, pre-control, and after control indicating elevation of bilirubin pre-control relative to baseline and creatinine post-control relative to pre-control and baseline. Values compared with repeat-measures ANOVA with ns, not significant, *p-value <0.05, and **p-value <0.005. Error bars represent standard error of the mean.

Ten of the eleven infected rhesus monkeys exhibited pulmonary hyperplasia, interstitial thickening, and fibrotic changes in the lung (**Figures 4A, B** and **Supplemental Table 2**). Abundant hemozoin was noted in both iRBCs and phagocytes in the interstitial areas and vasculature of infected parenchymal lung tissue (**Figure 4A**). Fibrotic changes in the lungs are highlighted in bright blue with Masson trichrome staining (**Figure 4B**). A less common but severe histological finding included mild to moderate pulmonary hemorrhage (**Figure 4A**). The liver showed periportal mononuclear infiltrate, Kupffer cell hyperplasia, and congestion in the hepatic sinuses (**Figure 4C**). Hemozoin was abundantly distributed in the vasculature as well in phagocytes in the sinuses. Neither hepatocellular necrosis nor cholestasis were observed (**Figure 4C**). Chronically infected monkeys exhibited similar pathological findings as that of acute infections, with the exception of one animal that had unique nodular infiltrates and material consistent with fibrous tissue deposition in the periportal areas of the liver (**Figure 4D**). The kidneys of all animals had glomerular hypercellularity. No glomerulonephritis was observed. Six animals displayed tubular degeneration, and five had interstitial inflammation (**Figure 4E** and **Supplemental Table 2**).

Remarkably, nine rhesus monkeys had moderate to severe stomach inflammation (**Figure 4F** and **Supplemental Table 2**). The duodenum, jejunum, and colon showed mild to moderate mucosal mononuclear infiltration, and mild edema was present in the colon (**Supplemental Table 2**). The tissues were assessed to provide semi-quantitative scores for statistical comparison (**Supplemental Table 2**), as performed in recent studies with *Saimiri boliviensis* and *M. fascicularis* infections with *P. vivax* and *P. knowlesi*, respectively (Peterson et al., 2019; Peterson et al., 2021). Though none of the tissues either prior to or after the rhesus monkeys controlled their infections exhibited any statistically significant changes, stomach (pre-control mean score = 3.44 ± 1.76; post-control mean score = 1.67 ± 1.52), liver (pre-control mean score = 4.62 ± 0.92; post-control mean score = 5.33 ± 0.58), and lung (pre-control mean score = 3.50 ± 0.76; post-control mean score = 5.00 ± 1.73) tended to exhibit the highest scores (**Figure 4G**). Consistent with the observation that chronically infected rhesus monkeys suffered similar organ pathology to rhesus monkeys with shorter courses of infection, pairwise comparisons revealed no statistically significant difference of organ pathology between those necropsied prior to parasitemic control and those necropsied afterwards (**Figure 4G**). Mixed-effects modeling revealed that neither necropsy day nor parasitemia affected the score (**Supplemental Table 3**); however, the tissue itself did (p-value = 7.78 x 10^-12^).

Differences in systemic measurements of liver function [i.e., alanine transaminase (ALT), aspartate aminotransferase (AST), and total bilirubin], were also assessed by comparing values obtained from baseline time points prior to infection (days -49 to -3), those taken in the pre-control period (determined to be prior to days 31-35 post infection), and in the controlled period (**Figures 4H–K**). Neither ALT nor AST values were significantly elevated from baseline levels (**Figures 4H, I**). Bilirubin was significantly elevated from baseline in the pre-control period (p-value = 0.04) (**Figure 4J**). Systemic kidney function was also assessed. Creatinine was significantly elevated post-parasitemic control relative to baseline (p-value = 1.90 x 10^-3^) (**Figure 4K**).

### Parasite Accumulation in Rhesus Monkey Tissue Vasculature During Acute *P. knowlesi* Infection Parallels Histopathology

**Figure 5:**
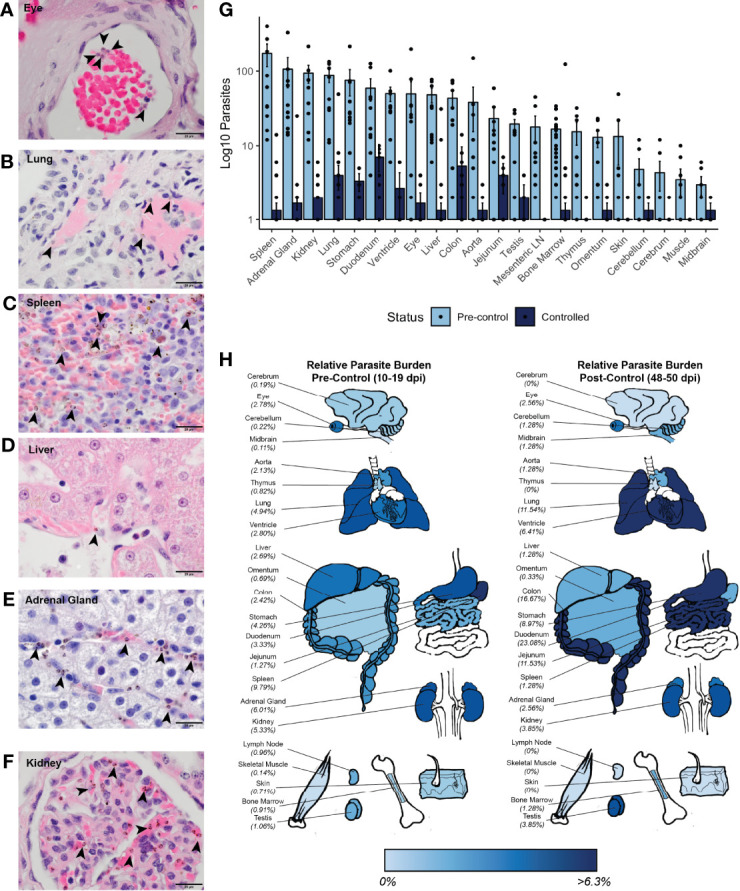
Parasite tissue burden in *P. knowlesi*-infected rhesus monkeys. Examples of iRBCs at 1000x (oil immersion) in H&E-stained tissue sections of **(A)** eye, **(B)** lung, **(C)** spleen, **(D)** liver, **(E)** adrenal gland, and **(F)** kidney. Parasitized RBCs are indicated with black arrowheads. **(G)** Mean log_10_-transformed parasite counts in 10 high-powered fields for 22 different tissues in pre-control and post-control monkeys. Error bars represent standard error of the mean. **(H)** Heat map of relative parasite densities indicating parasite burden in pre-control and controlled infections.

Parasite accumulation in the tissue vasculature prior to and after control of parasitemia in each of the infected rhesus was evaluated to assess possible associations with organ histopathology. The number of iRBCs in 10 high-power fields (HPFs) was enumerated in 22 tissues to provide approximate iRBC densities within each tissue (**Supplemental Table 4**). Representative images of parasites identified in selected tissues are shown in **Figures 5A–F**. Consistent with the lower, on average, circulating parasitemias after the monkeys were able to control their parasitemias (**Figure 1**), there was less accumulation of iRBCs at these time points relative to earlier time points (**Figure 5G** and **Supplemental Table 4**). Hierarchical multivariate analysis revealed that counts were significantly associated with the circulating parasitemia and tissue (**Supplemental Table 5**).

To determine if there may be a preference for iRBC accumulation in specific tissues, the distributions of the iRBCs in the tissues was compared against a uniform distribution using a χ^2^ test. This analysis indicated that the distribution of the iRBCs in the tissues was not evenly distributed prior to control of parasitemia, consistent with a preference for iRBC accumulation in specific tissues (**Figure 5G** and **Supplemental Table 4**). To assess if there were differences in iRBC accumulation in the tissues irrespective of their different parasitemias, relative parasite densities were 1) determined by normalizing the iRBC load, and 2) displayed in a heat map (**Figure 5H**). Tissues with the highest parasite accumulation included the spleen, colon, stomach, lung, liver, heart, and kidney prior to parasitemia control. Post-control, the spleen is less evident as a reservoir, but the GI tract, lungs, and heart remain predominant sites for parasite accumulation (**Figure 5H**).

Multiple analyses were used to assess if the accumulation of parasites in the tissues was associated with tissue damage, as quantified by histopathology scores (**Supplemental Table 2**). First, univariate analysis using Spearman’s correlation was performed between tissue score and iRBC density. Parasite iRBC burden positively correlated with tissue score (ρ = 0.308, p-value = 4.66x10^-5^), with a stronger correlation prior to monkey control of parasitemia (ρ = 0.432, p-value = 2.32x10^-5^). Next, a hierarchical multiple linear regression analysis was performed to determine which factors affected tissue scores the most (**Supplemental Table 5**). A model considering only iRBC tissue burden did not result in a linear relationship (adjusted R^2^ = 0.060, p-value = 0.0008), however, when combined with the tissue type, the relationship was essentially linear (adjusted R^2^ = 0.856, p-value < 2.20 x 10^-16^). The contribution of the iRBC tissue burden to the model remained significant after the addition of tissue type (p-value = 5.20 x 10^-15^). A model including an interaction term between the tissue iRBC burden and tissue histopathology score did not improve the fit of the model (R^2^ = 0.856, p-value = 2.20 x 10^-16^), and the interaction term was not a significant contributor. No multicollinearity was noted. Parasitemia at necropsy and cumulative parasitemia were not significant contributors. Thus, although there is a relationship between parasite accumulation and overall histopathology within tissues during acute infection, parasite accumulation alone does not determine the degree to which tissues are damaged.

### *P. knowlesi* Accumulation in the Rhesus Monkey Tissue Vasculature May be Due to the Expression of SICA Variant Antigens

**Figure 6:**
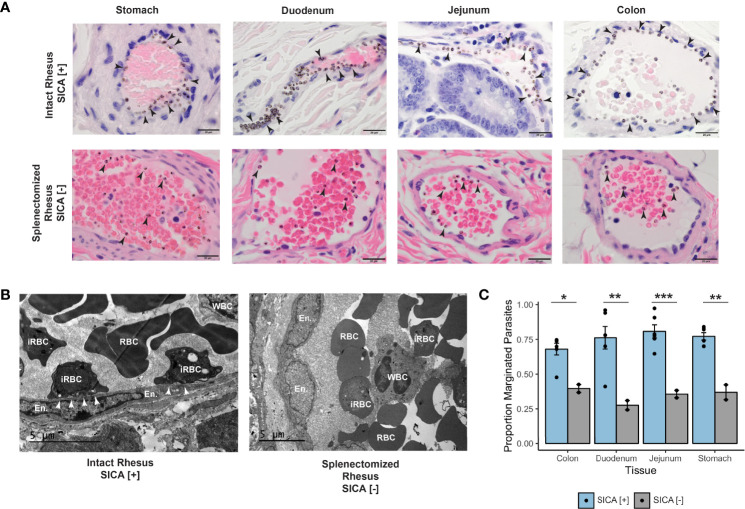
Margination, SICA protein expression, and binding of *P. knowlesi*-infected RBCs. **(A)** Top: infected iRBCs were noted to form a very clear margination pattern in rhesus monkeys infected with SICA-expressing parasites in the tissues of the gastrointestinal tract (1000x). Bottom: The margination phenotype is lost when monkeys are infected with SICA protein phenotypic negative parasites. Infected RBCs are indicated with black arrowheads (1000x). **(B)** Top: TEM of representative rhesus monkey duodenum samples shows contact between SICA[+] iRBCs and the endothelium (white arrowheads). Bottom: No contact was observed between SICA[-] iRBCs. **(C)** Quantification of the percentage of iRBCs located on the periphery of vessels in H&E-stained sections revealed significant differences between SICA[+]-infected and SICA[-]-infected splenectomized monkeys. Statistical comparison analysis was conducted with FDR-corrected Student’s t-tests comparing SICA [-] and SICA [+] infected tissues, by tissue (*p-value <0.05, **p-value<0.005, ***p-value<0.0005). Error bars represent standard error of the mean.

Small mucosal and submucosal blood vessels throughout the GI tract were observed to be packed with iRBCs in five of six pre-control monkeys at the acute phase of infection (**Figure 6A**, top row). Most dramatically, the iRBCs appeared to stud the endothelium of larger vessels, suggesting cytoadhesion of the iRBCs at the vascular interface (**Figure 6A**, top row). These ‘marginating’ iRBCs, visualized directly adjacent to endothelial cells, were evaluated by examining duodenal tissue samples by TEM (**Figure 6B**, left panel). Indeed, this analysis suggested direct interactions of parasitized RBCs with the endothelium.

Despite the presence of trophozoites and schizonts on blood smears, the parasite kinetics showed an undulating saw-tooth pattern consistent with cytoadhesion and sequestration of the maturing T/S-iRBC forms (**Supplemental Figures 1**–**4**; blood smears were performed twice daily during the acute period of the infections, when the parasitemias were rising (Taliaferro and Taliaferro, 1949)). This pattern reflects fewer parasites present as schizonts in the peripheral blood at the 8 AM time point than would be the case if all the developing ring-stage parasites seen in the afternoon the day before were still present in the circulation. These results are consistent with the cytoadhesion and sequestration of some but not all *P. knowlesi* iRBCs that constitute the blood-stage infections.

Since *P. falciparum* iRBC cytoadhesion is mediated by *Pf*EMP1surface-expressed variant antigens (reviewed in Lee et al., 2019), an experiment was performed to test the hypothesis that the related SICA proteins may likewise mediate the *P. knowlesi* iRBC margination phenotype. Two splenectomized rhesus monkeys were infected with SICA[-] parasites, and the GI tissues were collected and examined in the same manner as the SICA[+] infected animals. The percentage of iRBCs marginated within 350 vessels was calculated from the stomach, duodenum, jejunum, and colon and compared between splenectomized rhesus infected with SICA[-] parasites and the spleen-intact rhesus infected with SICA[+] parasites. In stark contrast to the iRBC margination observed in the tissues of the spleen-intact rhesus infected with SICA[+] parasites, there was no comparable margination of the SICA[-] iRBCs observed by light microscopy in the GI tissues from the splenectomized rhesus (**Figure 6A**, bottom row), and no endothelial cell contact was observed by TEM (**Figure 6B**, right panel). In agreement with the microscopy data, the percentage of iRBCs appearing to be marginated in the digestive tract (stomach, duodenum, jejunum, and colon) was significantly reduced in the rhesus infected with SICA[-] compared to SICA[+] parasites (**Figure 6C**). This was the case despite much higher parasitemias in the SICA[-] compared to SICA[+] infected animals. These analyses support the premise that, comparable to *Pf*EMP-1-endothelial cell interactions, a subpopulation of *P. knowlesi* iRBCs cytoadheres and sequesters, in part, due to the expression of SICA proteins on the surface of *P. knowlesi*-iRBCs.

## Discussion

This study determined clinical features of infection and host-parasite interactions that underpin disease severity in *P. knowlesi* infections in rhesus macaques, providing insights into mechanisms of severe illness in human *Plasmodium* infections. In the absence of treatment, overwhelming parasitemia is the main cause of death for rhesus macaques infected with *P. knowlesi* (reviewed in Howard, 1984). Importantly, rhesus monkeys in this study became febrile by day 8 post-inoculation of sporozoites, one day on average later than kra monkeys (Peterson et al., 2021), which, as expected, showed natural resilience to experimental *P. knowlesi* infections without treatment (Knowles and Gupta, 1932; Garnham, 1966; Collins et al., 1992). This ‘one-day’ difference in fever response was apparent even though both macaque species developed blood-stage parasitemias within a typical time frame of 6-7 days from the time of sporozoite inoculation. The later febrile episodes in the rhesus monkeys suggest that they were less able or unable to detect and mount appropriate, timely systemic inhibitory responses to control the blood-stage parasites. Since *P. knowlesi*-iRBCs multiply approximately 10-fold every 24 hours, this seemingly unremarkable ‘one-day delay’ in rhesus monkey responsiveness is significant. The delayed containment of parasitemia resulted in apparent prolonged inflammation in the rhesus compared to recovery processes in the kra monkeys (Gupta et al., 2021; Gupta et al., 2022).

Bilirubinemia has been associated with severe *P. knowlesi* malaria in humans (Barber et al., 2013), and it was mildly elevated in the rhesus monkeys in this study prior to them controlling their parasitemia. Fractionated bilirubin measurements were unavailable, however, and given that there was no histological evidence for cholestasis or other hepatic injury, the elevation in total bilirubin may reflect increased hemolysis. The anemia in the rhesus monkeys was mostly mild and may correspond to the relatively modest increase in serum bilirubin observed. Neither AST nor ALT was significantly different from baseline, however an increase in AST was observed. ALT and AST are both measures of cell integrity, with ALT being more specific to the liver. An elevation of AST without a concurrent elevation of ALT in concert with the absence of hepatocyte necrosis is consistent with a picture of nonspecific tissue stress caused by an infection (Giannini et al., 2005). Despite notable histopathology in the kidneys, both prior to and after parasitemic control, no significant elevation in creatine was observed until after control of the infection.

After subcurative antimalarial medication was administered to the acutely infected rhesus, to ensure their survival and to establish chronic infections, the observed infection-based decreased hemoglobin and platelet levels rebounded rapidly in the animals. The relatively mild anemia and thrombocytopenia occurring in the animals are consistent with expectations based on human cases after antimalarial treatment (Daneshvar et al., 2009). Insufficient erythropoiesis has been reported in rhesus macaques infected with *P. cynomolgi* and *P. coatneyi*, as well in rodents and humans infected with *Plasmodium* parasites (Chang et al., 2004; Chang and Stevenson, 2004; Perkins et al., 2011; Moreno et al., 2013; Thawani et al., 2014; Joyner et al., 2016; Tang et al., 2017; Cordy et al., 2019). Likewise, here, the BM response was blunted in the face of *P. knowlesi* infection, until treatment was provided. Interestingly, in stark contrast, the BM response was timely in kra monkeys infected with *P. knowlesi*, with the kra monkey BM responding naturally to the infection without treatment (Peterson et al., 2021). This is consistent with their faster overall recovery from infection compared to the rhesus, as indicated by transcriptomics (Gupta et al., 2021; Peterson et al., 2021; Gupta et al., 2022). Future determinations of the mechanism(s) by which hemoglobin levels in rhesus monkeys stabilize after subcurative treatment may lead to a better understanding of malarial anemia in relation to patient care.

Much remains to be learned about the specific systemic host-parasite interactions and biological, immunological, and metabolic cascades that influence the ultimate pathology and disease course of malaria in both humans and NHPs. Tissue data obtained from autopsy and necropsy procedures, respectively, to better understand malaria pathology in the context of clinical data and the clinical spectrum of the disease, can be powerful, providing a holistic assessment far beyond what can be achieved with either cross-sectional samples or multiple time points during infections. Previous studies have reported the presence of *P. knowlesi* iRBCs in various tissues from infected rhesus monkeys (Miller et al., 1971b; Anderios et al., 2010). The data presented here expand upon that knowledge by quantitatively determining that *P. knowlesi* iRBCs are preferentially found in specific organs like the spleen, adrenal gland, lung, kidney, and the organs of the GI tract. Similar to prior studies with *P. vivax* infections of *S. boliviensis* monkeys (Peterson et al., 2019), a modest relationship was identified here between the rhesus histopathology scores and *P. knowlesi* iRBC accumulation in these tissues. While *P. knowlesi* iRBC accumulation in the tissues may directly or indirectly cause histopathology, our data suggest that it is neither necessary nor sufficient. Instead, host responses are likely the main contributors to the observed tissue damage. This hypothesis, which warrants further exploration, is in fact consistent with tissue-damage studies that have directly shown that *P. falciparum*-iRBC accumulation in the brain is not necessarily the main driver of cerebral malaria (Seydel et al., 2006; Milner et al., 2014; Milner et al., 2015; Coban et al., 2018).

Acute *Plasmodium* infections from multiple species – whether in NHPs or humans – are known to cause damage to tissues and vital organs, which can contribute to clinical complications and death (Spitz, 1946; Spangler et al., 1978; Cox-Singh et al., 2010; Lacerda et al., 2012; Milner et al., 2014). Therefore, it was anticipated in this study that escalating parasitemias in rhesus would cause tissue damage that, at least in part, would contribute to the inability of this species to survive infection without treatment (Spangler et al., 1978). Surprisingly, the histopathology observations during the acute phase of the rhesus infections proved to be comparable to those reported previously for *P. knowlesi*-infected kra monkey tissues (Peterson et al., 2021) and dramatic pathological findings in vital organs (*e.g*., lung, liver, kidney) were not identified. However, gastritis was specifically noted in the rhesus stomach tissue samples acquired from necropsy performed at the time of the acute infections. This finding is noteworthy from a clinical perspective because gastritis and other GI complications and clinical manifestations occur frequently in humans with *P. knowlesi* malaria (Cox-Singh et al., 2008; Barber et al., 2013).

Striking observations presented here include microscopic evidence for sequestration of *P. knowlesi* SICA[+] iRBCs with presumptive cytoadhesion that was pronounced in the spleen-intact rhesus. Frank margination of the iRBCs was observed in the mucosal and submucosal vessels of the stomach, duodenum, jejunum, and colon tissues from these animals, and this phenotype was dramatic even with relatively low parasitemias (1-3%) at the time of necropsy. Rheological changes and ‘sludging,’ as reported previously in rhesus infection studies, in part explain capillary congestion with the *P. knowlesi*-iRBCs (Knisely et al., 1945; Knisely et al., 1964; Miller et al., 1971a; Barber et al., 2018). Yet, the clear predilection shown here for iRBCs at the margins of venules over the lumen implicates iRBC endothelial interaction(s) and cytoadhesion as an apparent mechanism.

A few studies have examined cytoadhesion of *P. knowlesi* T/S-iRBCs using *ex vivo* (Fatih et al., 2012) and *in vitro* (Lee et al., 2022) cultivated parasites, leaving questions as to the parasite ligand(s) involved and the number and types of possible host receptor specificities. Critically, *in vitro* cultivated *P. knowlesi* iRBCs have been shown to lose expression of the *SICAvar* gene family, and these were classified in the ‘off state’ with regards to *SICAvar* expression (Lapp et al., 2015). Expression of the *SICAvar* gene family and the encoded SICA proteins were shown to be downregulated to the ‘off state’ in iRBCs from splenectomized hosts, bringing emphasis to the importance of the *in vivo* splenic environment for regulating SICA expression (Lapp et al., 2013) and ultimately virulence of *P. knowlesi* infections (Barnwell et al., 1983b). In turn, experimental systems to test for receptor-ligand interactions may require *ex vivo* parasites obtained from spleen-intact animals. Our preliminary findings in this area (unpublished data), as others (Fatih et al., 2012; Lee et al., 2022), suggest levels of complexity on par with *P. falciparum*. Therefore, sorting out the precise receptors, ligands, and interactions that support *P. knowlesi* cytoadhesion and sequestration will not be an easy task.

SICA proteins have been considered prime candidates mediating possible adhesive interaction(s), due to their commonalities with *Pf*EMP1, including iRBC surface localization and multiple diverse externalized cysteine-rich domains (Al-Khedery et al., 1999; Korir and Galinski, 2006); these features are hallmarks of *Pf*EMP1 that account for *P. falciparum*’s selective T/S-iRBC cytoadhesion characteristics and sequestration (reviewed in Howard, 1984; Smith, 2014). Here, tissues from splenectomized rhesus monkeys infected with SICA[-] parasites lacked the frank margination phenotype observed in the tissues from spleen-intact monkeys infected with SICA[+] parasites. This observation strongly supports the premise that SICA proteins mediate the adhesion of the iRBCs to the endothelium and provides further impetus for studying the SICA[+] and SICA[-] parasites in parallel infections to better understand the pathological mechanisms and biological implications of iRBC adherence to the endothelium. SICA protein expression at the surface of *P. knowlesi*-iRBCs is associated with virulence in rhesus and *SICAvar* gene family expression is somehow regulated by the spleen (Barnwell et al., 1983a; Lapp et al., 2013), as is *Pf*EMP1 variant antigen gene and protein expression in *Saimiri* monkeys and humans (David et al., 1983; Hommel et al., 1983; Bachmann et al., 2009).

Nonetheless, as for *Pf*EMP1, it is worth noting that the biological function of SICA in the context of the iRBC membrane remains unknown (reviewed in Howard, 1984; Galinski et al., 2018). In this context, it is important to recognize that cytoadhesion – as proposed to avoid circulation through the spleen – is not necessarily (nor likely) the sole function of either of these proteins (reviewed in Howard, 1984). It would be a breakthrough if an essential iRBC function were to be discovered for the *Pf*EMP-1 and SICA proteins, commensurate with these parasite species’ evolved strategies to maintain numerous *var/SICAvar* family members to ensure survival. Clearly, as is known for *P. knowlesi* and most species of *Plasmodium* where all blood-stage forms circulate, avoidance of the spleen does not fit as the main function of potentially adhesive surface-exposed proteins. In fact, all other *Plasmodium* species known to infect humans do not have comparable *var/SICAvar* genes (*P. vivax*, *P. malariae*, *P. ovale*, *P. cynomolgi*) (Tachibana et al., 2012; Rutledge et al., 2017; Imwong et al., 2018) and all their iRBC developmental life cycle stages circulate, seemingly unaffected by passage through the spleen (or potentially even benefiting from intra-splenic interactions (reviewed in Kho et al., 2021; Fernandez-Becerra et al., 2022). All iRBC developmental forms of these species circulate despite *P. malariae* and *P. ovale* sharing knobby iRBC surface phenotypes (Matsumoto et al., 1986; Li et al., 2010) that are morphologically similar to those in *P. falciparum* infections (Gruenberg et al., 1983; Aikawa et al., 1996). The iRBCs of each of these species differ from *P. knowlesi*, which has occasional indentations in the form of caveolae peppering the iRBC and no knobby protrusions (Liu et al., 2019). In fact, *P. knowlesi* is the simplest of them all, having neither knobs nor caveolae vesicle complexes, which are especially abundant at the iRBC surface of *P. vivax-* and *P. cynomolgi-*iRBCs (Aikawa et al., 1975; Akinyi et al., 2012). The simplicity, by comparison, of the *P. knowlesi* iRBC membrane, even compared to the phylogenetically related ‘knobby, variant and sequestering’ simian malaria parasites *P. fragile* and *P. coatneyi* (Garnham, 1966; Fremount and Miller, 1975; Handunnetti et al., 1987; Nakano et al., 1996; Collins et al., 2006; Chien et al., 2016), makes it an attractive parasite model for understanding the essential features of *Plasmodium*-iRBC parasitism and their evolution.

In sum, this study reports a systematic study of *P. knowlesi* sporozoite-infected rhesus monkeys that expands our understanding of pathogenic features of severe disease and makes comparisons with comparable data from kra monkeys that support resilience to *P. knowlesi* malaria (Peterson et al., 2021). Multiple physiological and immunological responses were found to be associated with pathology or resilience. Additionally, an association of SICA variant proteins with cytoadhesion and sequestration came to light in comparisons of SICA[+] and SICA[-] parasites in tissues obtained from spleen-intact and splenectomized rhesus, respectively. Studies using multiple NHP models in biomedical research have been beneficial for several decades towards understanding HIV pathogenesis, specifically with the comparison of SIV-infected rhesus macaques and sooty mangabeys in relation to the development of AIDS (Bosinger et al., 2009; Palesch et al., 2018). Correspondingly, a two-host animal model system that includes rhesus and kra monkeys and longitudinal infections is now poised for significant advances towards understanding host-parasite interactions, cascades and networks. Host mechanistic differences in physiology, immune responses, and pathogenesis, can be unravelled to better understand malaria severity and resilience against *P. knowlesi* and potentially other species of *Plasmodium* that can infect these species.

## Consortium Membership

MaHPIC members participating in discussions at the time of the planning, implementation, or analysis of this project include: Dave C. Anderson, Ferhat Ay, Cristiana F. A. Brito, John W. Barnwell, Megan DeBarry, Steven E. Bosinger, Jung-Ting Chien, Jinho Choi, Anuj Gupta, Jay C. Humphrey, Chris Ibegbu, Xuntian Jiang, Dean P. Jones, Nicolas Lackman, Tracey J. Lamb, Frances E.-H. Lee, Karine Gaelle Le Roche, Shuzhao Li, Esmeralda V.S. Meyer, Diego M. Moncada-Giraldo, Dan Ory, Jan Pohl, Saeid Safaei, Ignacio Sanz, Maren Smith, Gregory Tharp, ViLinh Tran, Elizabeth D. Trippe, Karan Uppal, Susanne Warrenfeltz, Tyrone Williams, Zerotti L. Woods.

## Data Availability Statement

The clinical datasets generated and analyzed for this study can be found in the NIAID Bioinformatics Resource Center Database, PlasmoDB.org, https://plasmodb.org/plasmo/app/static-content/PlasmoDB/mahpic.html (Aurrecoechea et al., 2009; Amos et al., 2022).

## Ethics Statement

The animal study was reviewed and approved by Emory University’s Institutional Animal Care and Use Committee (IACUC) and the Animal Care and Use Review Office (ACURO) of the US Department of Defense.

## Author Contributions

Conceived and designed the experiments: MP, CJ, JB, JW, LF, RT, JK, AM, SG, EV, JG, RC, MG, and members of the MaHPIC-Consortium. Performed the experiments: MP, CJ, JB, JW, MC-M, CS, LF, WC, JJ, SL, SS, AH, DM, EK. Performed data analysis: MP, CJ, JB, LF, SG, JG. Interpreted the data analysis: MP, CJ, RC, MG and members of the MaHPIC-Consortium. Managed and led validation and quality control of datasets for clinical and telemetry results and deposited the data and metadata: MN, JD, JK. Generated the figures: MP, CJ, LF, SG. Wrote the paper: MP, MG. Provided manuscript editorial contributions: CJ, SL, LF, JD, RT, JK, AM, SG, EV, JG, RC. All authors reviewed the manuscript. All authors read and approved the final manuscript.

## Funding

This project was funded in part by the National Institute of Allergy and Infectious Diseases; National Institutes of Health, Department of Health and Human Services, which established the MaHPIC [Contract No. HHSN272201200031C; MG], the NIH Office of Research Infrastructure Programs/OD P51OD011132, the Defense Advanced Research Program Agency and the US Army Research Office *via* a cooperative agreement [Contract No. W911NF16C0008; MG], which funded the Technologies for Host Resilience-Host Acute Models of Malaria to study Experimental Resilience (THoR’s HAMMER) consortium.

## Conflict of Interest

The authors declare that the research was conducted in the absence of any commercial or financial relationships that could be construed as a potential conflict of interest.

## Publisher’s Note

All claims expressed in this article are solely those of the authors and do not necessarily represent those of their affiliated organizations, or those of the publisher, the editors and the reviewers. Any product that may be evaluated in this article, or claim that may be made by its manufacturer, is not guaranteed or endorsed by the publisher.

## Abbreviations

ALT, Alanine transaminase; AST, Aspartate aminotransferase; BM, Bone marrow; E, Experiment; CBC, Complete blood count; GI, Gastrointestinal; H&E, Hematoxylin and Eosin; iRBC, Infected red blood cell; MaHPIC, Malaria Host-Parasite Interaction Center; NHP, Nonhuman primate; *Pf*EMP-1, *P. falciparum* erythrocyte membrane protein-1; RBC, Red blood cell; RPI, Reticulocyte Production Index; SICA, Schizont-Infected Cell Agglutination; SICA[+], SICA positive; SICA[-], SICA negative; *SICAvar*, Schizont-Infected Cell Agglutination variant protein/antigen gene; Spx, Splenectomy; TEM, Transmission electron microscopy; T/S, Trophozoite/Schizont; *var*, variant antigen gene.
