# Supplementary material for: Plasmodium knowlesi Cytoadhesion Involves SICA Variant Proteins
Source: Front Cell Infect Microbiol. 2022 Jun 23;12:888496. doi: 10.3389/fcimb.2022.888496 (PMC9260704; doi:10.3389/fcimb.2022.888496)
Supplement: Supplementary file 1 [file DataSheet_1.pdf]

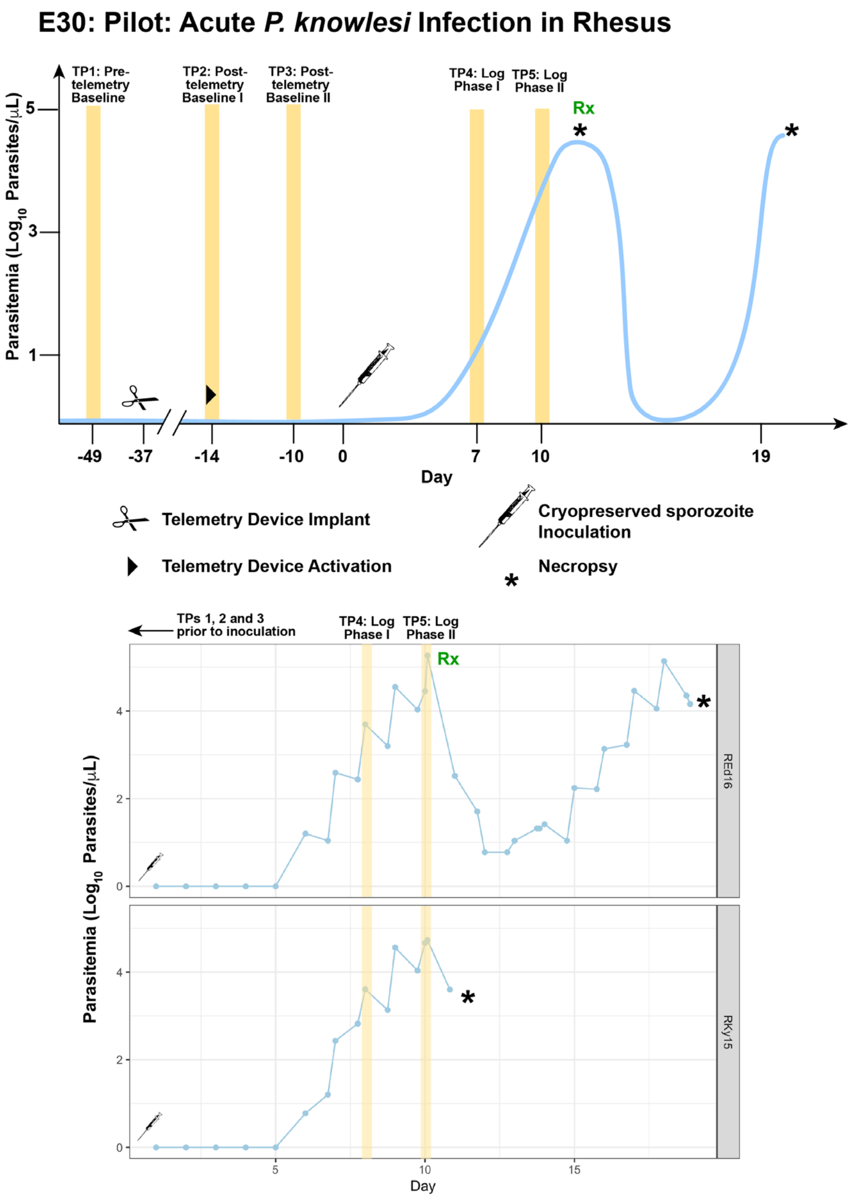

Supplemental Figure 1: E30 Experimental Schematic and Parasitemia Curve. **Top**: An idealized schematic illustrating the design and termination of E30, a pilot experiment which included two rhesus monkeys surgically implanted with telemetry technology. One animal was designated to be necropsied after the parasitemia reached at least 1% after patency, and the other was to be sub-curatively treated with artemether and necropsied when the parasitemia reached at least 1% during the first recrudescence. **Bottom**: The parasitemia curves with time points and necropsies indicated. TP = time point; Rx = administration of subcurative artemether treatment. Monkey code provided in gray box at right of plot.
