# Supplementary material for: Plasmodium knowlesi Cytoadhesion Involves SICA Variant Proteins
Source: Front Cell Infect Microbiol. 2022 Jun 23;12:888496. doi: 10.3389/fcimb.2022.888496 (PMC9260704; doi:10.3389/fcimb.2022.888496)
Supplement: Supplementary file 2 [file DataSheet_2.pdf]

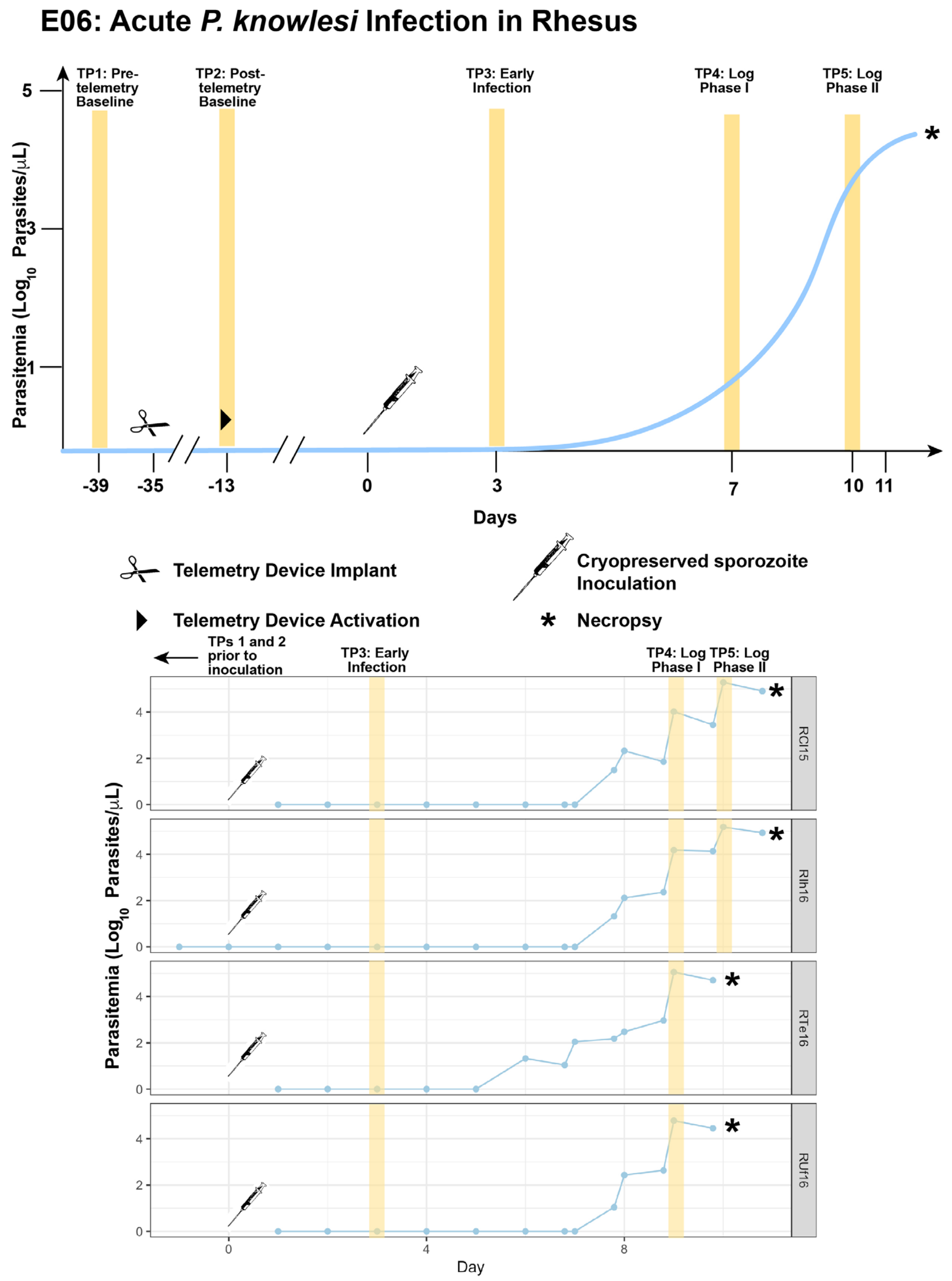

Supplemental Figure 2: E06 Experimental Schematic and Parasitemia Curve. **Top**: An idealized schematic illustrating the design and termination of E06, an experiment which included four rhesus monkeys surgically implanted with telemetry technology and terminated after the infection became patent and reached at least 1% parasitemia (40,000 parasites/µL). **Bottom**: The parasitemia curves with time points and necropsies indicated. TP = time point. Monkey code provided in gray box at right of plot.
