# Supplementary material for: Plasmodium knowlesi Cytoadhesion Involves SICA Variant Proteins
Source: Front Cell Infect Microbiol. 2022 Jun 23;12:888496. doi: 10.3389/fcimb.2022.888496 (PMC9260704; doi:10.3389/fcimb.2022.888496)
Supplement: Supplementary file 3 [file DataSheet_3.pdf]

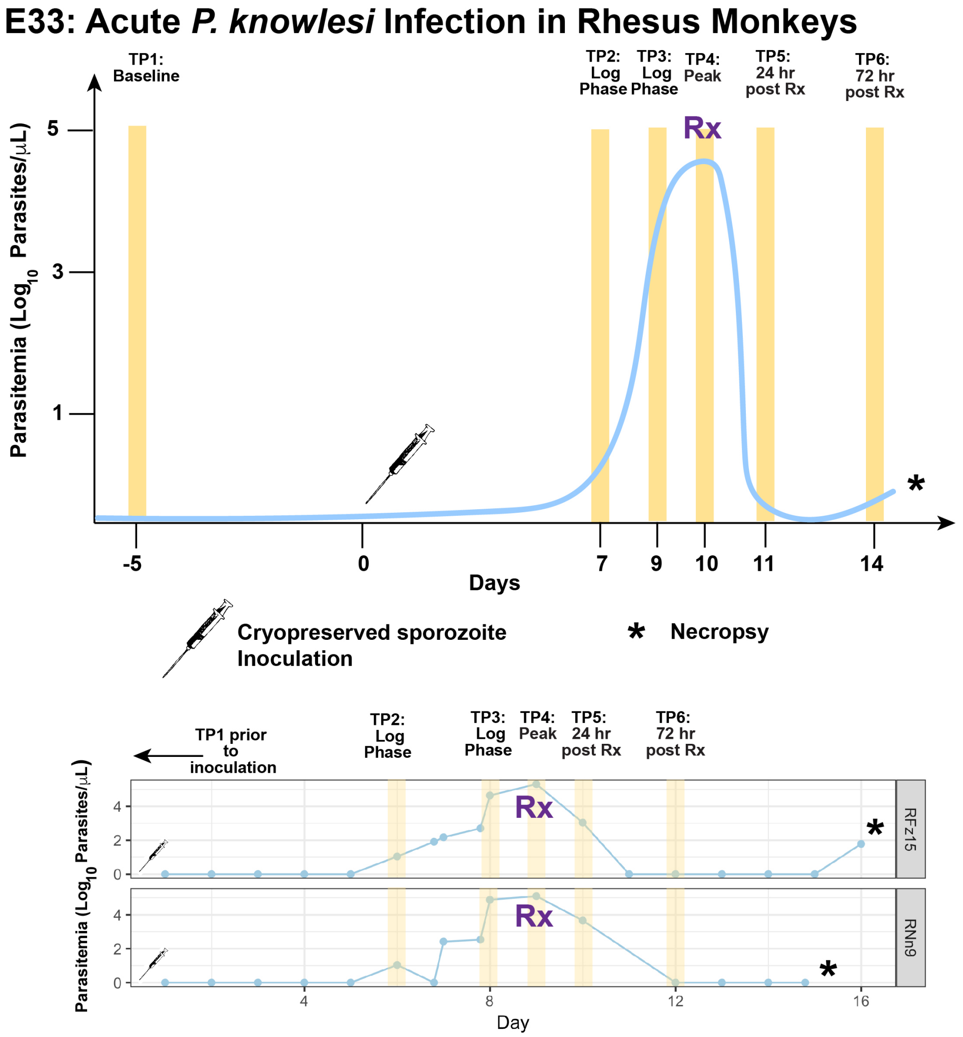

Supplemental Figure 3: E33 Experimental Schematic and Parasitemia Curve. **Top**: An idealized schematic illustrating the design and termination of E33, an experiment which included two rhesus monkeys sub-curatively treated with chloroquine and terminated 6-7 days post treatment. **Bottom**: The parasitemia curves with time points and necropsies indicated. TP = time point; Rx indicates subcurative treatment with chloroquine. Monkey code provided in gray box at right of plot.
