# Supplementary material for: Plasmodium knowlesi Cytoadhesion Involves SICA Variant Proteins
Source: Front Cell Infect Microbiol. 2022 Jun 23;12:888496. doi: 10.3389/fcimb.2022.888496 (PMC9260704; doi:10.3389/fcimb.2022.888496)
Supplement: Supplementary file 4 [file DataSheet_4.pdf]

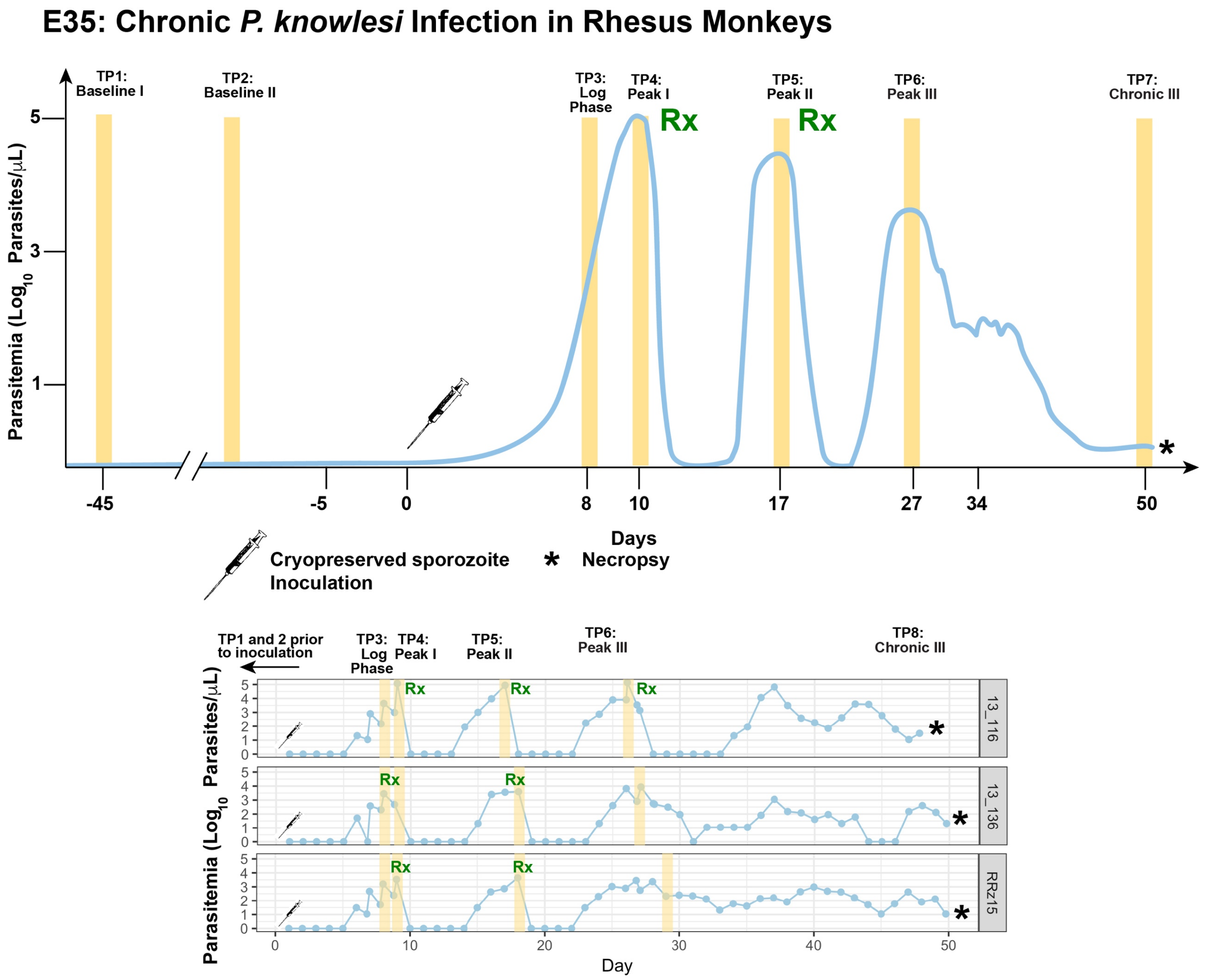

Supplemental Figure 4: E35 Experimental Schematic and Parasitemia Curve. **Top**: An idealized schematic illustrating the design and termination of E35, an experiment which included three rhesus monkeys sub-curatively treated with artemether at each recrudescent peak until the animals were able to control infection without intervention, then terminated at 48-50 dpi. **Bottom**: The parasitemia curves with time points and necropsies indicated. TP = time point; Rx indicates subcurative treatment with artemether. Monkey code is indicated in gray box to the right of plot.
