# Supplementary material for: Plasmodium knowlesi Cytoadhesion Involves SICA Variant Proteins
Source: Front Cell Infect Microbiol. 2022 Jun 23;12:888496. doi: 10.3389/fcimb.2022.888496 (PMC9260704; doi:10.3389/fcimb.2022.888496)
Supplement: Supplementary file 5 [file DataSheet_5.pdf]

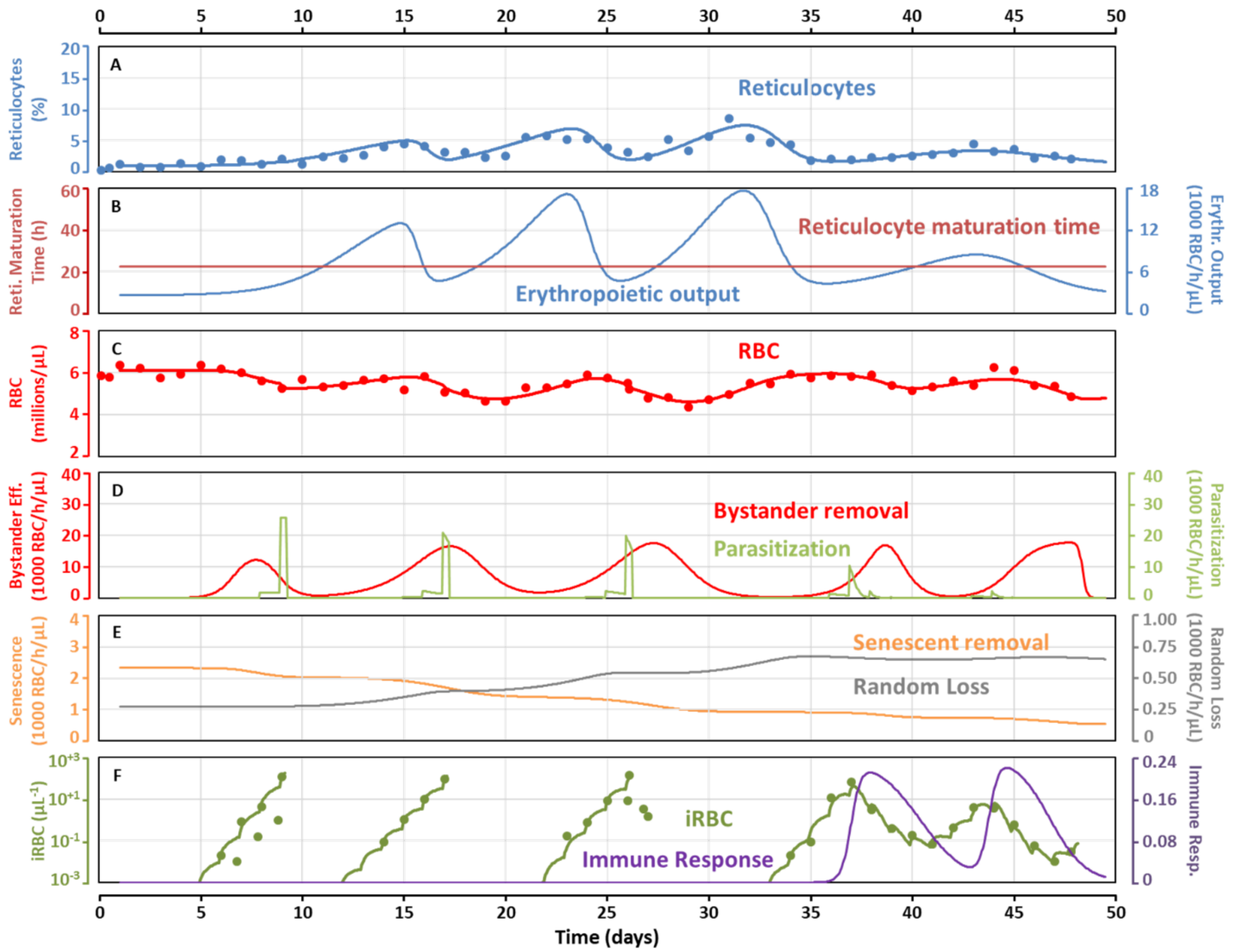

Supplementary Figure 5. Characterization of the dynamics of the RBC removal and production processes in a representative rhesus monkey (13_116) from Experiment 35 over a 50-day period of infection with *P. knowlesi* after inoculation with sporozoites. To quantify the hemodynamic processes, a computational model was used as previously developed to faithfully track the blood dynamics during a *Plasmodium* infection (Fonseca et al. 2018). The model was formulated as a set of discrete recursive equations, where the pools of reticulocytes, RBCs, and iRBCs were stratified into hourly age classes. The model assumes that reticulocytes are released from the bone marrow with a certain age and rate, circulate for a day, and then mature into RBCs. Model results (lines) are superimposed on experimental data (symbols). The experimental results included are: numbers of circulating reticulocytes (A), mature RBCs (C), and infected RBCs (F), from which the model allowed the quantification of different causes of RBC removal (D and E). The RBCs of *M. mulatta* normally die after about 100 days due to senescence (Fonseca et al. 2016), and by "random" effects, such as shear stresses (E). During *Plasmodium* infections, some of the healthy RBCs are infected by merozoites and destroyed when the parasites are released (parasitization, D) or lost to a bystander effect (D). Although the monkey in this example exhibited reasonably high parasitemias that led to some losses of RBCs by parasitization (D), and had to be treated 3 times (days 9, 17, and 26) to keep the parasitemia under control, it still mounted an appropriate hemodynamic response. Even though large numbers of RBCs were lost during the infection due to the bystander mechanism (D), the RBC profile (C) demonstrates that this monkey was

able to respond appropriately by increasing the erythropoietic output (B) and did not require early release of reticulocytes (B). not require early release of reticulocytes (B).

Fonseca, L. L., H. S. Alezi, A. Moreno, J. W. Barnwell, M. R. Galinski, and E. O. Voit. 2016. 'Quantifying the removal of red blood cells in *Macaca mulatta* during a *Plasmodium coatneyi* infection', *Malar J*, 15: 410.

Fonseca, L. L., C. J. Joyner, C. L. Saney, MaHPIC-Consortium, A. Moreno, J. W. Barnwell, M. R. Galinski, and E. O. Voit. 2018. 'Analysis of erythrocyte dynamics in rhesus macaque monkeys during infection with *Plasmodium cynomolgi*', *Malar J*, 17: 410.
