# Supplementary material for: Plasmodium knowlesi Cytoadhesion Involves SICA Variant Proteins
Source: Front Cell Infect Microbiol. 2022 Jun 23;12:888496. doi: 10.3389/fcimb.2022.888496 (PMC9260704; doi:10.3389/fcimb.2022.888496)
Supplement: Supplementary file 6 [file Table_1.docx]

| **Supplemental Table 1: Macaque Cohort and Experimental Summaries** | | | | | |
| --- | --- | --- | --- | --- | --- |
| **Cohort** (Experimental Number)^§^ | **Species** (n) | **Infection Type** | **Animal Codes** | **Brief Experimental Description**  (*Supplementary Figures 1-5 describe and show graphed summaries*) | **Telemetry**  **Implants** |
| **1** (E30) | *Macaca mulatta* (2) | Pilot Experiment: *P. knowlesi*  Acute Infection of  *M. mulatta* | REd16, RKy15 | Two *M. mulatta* were infected with cryopreserved *P. knowlesi* sporozoites, and blood and bone marrow samples were collected for analysis at pre-determined intervals. One was euthanized and necropsied for pathology analyses when its parasitemia had reached life-threatening levels, the other was subcuratively treated with artemether at that point, and then euthanized and necropsied at the time of the second rise in life-threatening parasitemia. | Yes  *Continuous telemetry data was collected*^†^ |
| **2** (E06) | *M. mulatta* (4) | Iterative Experiment:  *P. knowlesi*  Acute  Infection of  *M. mulatta* | RCl15, RIh16, RTe16, RUf16 | Four *M. mulatta* were infected with cryopreserved *P. knowlesi* sporozoites. Blood and bone marrow samples were collected for analysis at pre-determined times throughout the course of the infections. The animals were euthanized and necropsied for pathology analyses when their parasitemia had reached life-threatening levels. | Yes  *Continuous telemetry data was collected*^†^ |
| **3** (E33) | *M. mulatta* (2) | Iterative Experiment:  *P. knowlesi*  Acute Infections & Establishment of Chronic Infections | RFz15, RNn9 | Two *M. mulatta* were infected with cryopreserved *P. knowlesi* sporozoites. Blood and bone marrow samples were collected for analysis at pre-determined intervals. The rhesus monkeys were subcuratively treated with chloroquine as the parasites were rising to life-threatening levels to evaluate the systems biological response of these hosts. The animals were euthanized and necropsied for pathology analyses within 2-3 weeks of inoculation as the *M. mulatta* were in a post-subcurative treatment period. | No |
| **4** (E34) | *M. mulatta* (3) | Control  Data | RAa16, RBe16, RQs9 | Three *M. mulatta* were sacrificed to provide normal control samples for analysis in conjunction with samples from infected macaques. | No |
| **5** (E35) | *M. mulatta* (3) | Iterative Experiment:  *P. knowlesi*  Acute & Chronic  Infection | 13_116, 13_136, RRz15 | Three *M. mulatta* were infected with cryopreserved *P. knowlesi* sporozoites. Blood and bone marrow samples were collected for analysis at pre-determined intervals throughout the experiment. The *M. mulatta* were subcuratively treated with artemether when parasitemias reached above 1% to prevent the inevitable continued rapid parasitemia rise and death, and to induce chronicity. The animals were euthanized and necropsied for pathology analyses between days 48-50. | No |
| **6** (Spx) | *M. mulatta* (2) | Validation Experiment:  *P. knowlesi*  Acute Infection of splenectomized  *M. mulatta* | RBg14, RAd14 | Two splenectomized *M. mulatta* were infected with Pk1(A-)1- , SICA[-] infected RBCs to test the hypothesis that in the absence of SICA protein expression the iRBCs would no longer marginate in the vessels; *i.e.*, by H&E and TEM no longer show apparent adhesion to the vascular endothelium. | No |

**Supplemental Table 1.** **Summary of Macaque Cohorts and Experimental Conditions.** Six monkey cohorts are summarized, four that were experimentally infected with *P. knowlesi* sporozoites (E30, E06, E33, E35), one with *P. knowlesi* infected RBCs (Spx), and one control group (E34). The six cohorts are listed in the order that experiments using these animals were performed. These animals and their longitudinal infection designs were part of a systems biology program, with iterative cohort experimentation designed to satisfy the goals of those research programs. ^§^The non-sequential experimental numbering (E30, E06, E33, E34, and E35) reflects the experimental numbers assigned in the MaHPIC Laboratory Information Management System. ^†^Telemetry data were collected for temperature, blood pressure, heart rate and activity level (Brady *et al*., manuscript in preparation). Spx is an abbreviation for splenectomy.
