# Supplementary material for: Plasmodium knowlesi Cytoadhesion Involves SICA Variant Proteins
Source: Front Cell Infect Microbiol. 2022 Jun 23;12:888496. doi: 10.3389/fcimb.2022.888496 (PMC9260704; doi:10.3389/fcimb.2022.888496)
Supplement: Supplementary file 7 [file Table_2.docx]

|  | **Rhesus Monkey Code** | | | | | | | | | | |
| --- | --- | --- | --- | --- | --- | --- | --- | --- | --- | --- | --- |
| **Histopathology** | RCl15 | RIh16 | RUf16 | RTe16 | RKy15 | REd16 | RNn9 | RFz15 | RRz15 | 13_116 | 13_136 |
| **Pre-Control = P**  **Controlled = C** | P | P | P | P | P | P | P | P | C | C | C |
| **Colon** |  |  |  |  |  |  |  |  |  |  |  |
| *Inflammation* | 1 | 1 | 1 | 1 | 2 | 2 | 1 | 1 | 2 | 1 | 1 |
| *Edema* | 1 | 1 | 0 | 0 | 0 | 0 | 0 | 0 | 1 | 0 | 0 |
| **Duodenum** |  |  |  |  |  |  |  |  |  |  |  |
| *Inflammation* | 2 | 2 | 2 | 2 | 2 | 2 | 2 | 2 | 2 | 2 | 2 |
| **Jejunum** |  |  |  |  |  |  |  |  |  |  |  |
| *Inflammation* | 2 | 1 | 2 | 2 | 2 | 1 | 1 | 1 | 1 | 1 | 1 |
| **Stomach** |  |  |  |  |  |  |  |  |  |  |  |
| *Inflammation* | 3.5 | 3 | 3 | 3 | 3 | 4 | 2 | 1 | 0 | 2 | 3 |
| *Crypt Inflammation* | 0 | 0 | 0 | 1 | 3 | 0 | 0 | 0 | 0 | 0 | 0 |
| **Kidney** |  |  |  |  |  |  |  |  |  |  |  |
| *Inflammation* | 0 | 0 | 0 | 1 | 1 | 1 | 0 | 0 | 0 | 1 | 1 |
| *Hemorrhage* | 0 | 0 | 0 | 0 | 0 | 0 | 0 | 0 | 0 | 0 | 0 |
| *Tubular Degeneration* | 2 | 1 | 0 | 1 | 1 | 0 | 0 | 0 | 2 | 2 | 0 |
| *Glomerular Hypercellularity* | 2 | 1 | 1 | 1 | 2 | 2 | 1 | 1 | 2 | 1 | 1 |
| **Liver** |  |  |  |  |  |  |  |  |  |  |  |
| *Inflammation* | 2 | 2 | 2 | 2 | 3 | 3 | 2 | 2 | 2 | 2 | 3 |
| *Kupffer Cell Hyperplasia* | 3 | 2 | 2 | 2 | 3 | 3 | 2 | 2 | 3 | 3 | 3 |
| **Lung** |  |  |  |  |  |  |  |  |  |  |  |
| *Inflammation* | 0 | 0 | 0 | 0 | 0 | 0 | 0 | 0 | 0 | 0 | 0 |
| *Hemorrhage* | 0 | 0 | 0 | 3 | 2 | 0 | 0 | 0 | 2 | 1 | 2 |
| *Hyperplasia* | 1 | 2 | 1 | 0 | 1 | 1 | 1 | 1 | 1 | 1 | 1 |
| *Fibrosis* | 2 | 2 | 2 | 0 | 2 | 2 | 3 | 2 | 3 | 1 | 3 |
| **Left Cardiac Ventricle** |  |  |  |  |  |  |  |  |  |  |  |
| *Inflammation* | 0 | 0 | 0 | 0 | 0 | 0 | 0 | 0 | 0 | 0 | 0 |
| *Edema* | 0 | 0 | 0 | 0 | 0 | 0 | 0 | 0 | 0 | 0 | 0 |
| *Hemorrhage* | 0 | 0 | 0 | 0 | 0 | 0 | 0 | 0 | 0 | 0 | 0 |

**Supplemental Table 2. Semi-quantitative pathology scores for *Plasmodium knowlesi*-infected rhesus macaques.** Blinded and randomized slides were scored for the degree of damage or severity of different histopathological changes. Scores were then added to create a total tissue score for each animal. Tissues with no damage were assigned a score of zero.
