# Supplementary material for: Plasmodium knowlesi Cytoadhesion Involves SICA Variant Proteins
Source: Front Cell Infect Microbiol. 2022 Jun 23;12:888496. doi: 10.3389/fcimb.2022.888496 (PMC9260704; doi:10.3389/fcimb.2022.888496)
Supplement: Supplementary file 8 [file Table_3.docx]

**Supplemental Table 3: Linear Regression: Pathology Score v. Parasitemia, Necropsy Day, and Tissue**

|  | Estimate | Std. Error | t value | p-value | Significance |
| --- | --- | --- | --- | --- | --- |
| Intercept | 2.11 | 0.30 | 6.96 | 1.65x10^-9^ | *** |
| Parasitemia | 4.78x10^-6^ | 2.86x10^-6^ | 4.06 | 1.28x10^-4^ | * |
| Necropsy Day | 1.51x10^-2^ | 8.48x10^-3^ | 1.78 | 0.08 | NS |
| *Tissue* |  |  |  |  |  |
| Duodenum | -1.20 | 0.29 | -4.20 | 7.95x10^-5^ | *** |
| Jejunum | -0.75 | 0.29 | -2.61 | 0.01 | * |
| Kidney | -1.38 | 0.29 | -4.835 | 7.94x10^-6^ | *** |
| Liver | -0.11 | 0.29 | -0.39 | 0.70 | NS |
| Lung | 2.07 | 0.29 | 7.24 | 5.19x10^-10^ | *** |
| Stomach | 1.16 | 0.29 | 4.06 | 1.29x10^-4^ | *** |
| N = 76; df = 68; Adjusted R^2^ = 0.56; F-statistic = 13.3; p-value = 2.23x10^-11^ | | | | | |

**Supplemental Table 3. Linear regression model results for pathology score vs. parasitemia, necropsy day, and tissue.** Multiple linear regression was performed to determine which factors affected total pathology score, including the day the animals were necropsied, the parasitemia at necropsy, and the tissue itself. ***p-value < 0.0005, *p-value < 0.05, NS = not significant.
