# Supplementary material for: Plasmodium knowlesi Cytoadhesion Involves SICA Variant Proteins
Source: Front Cell Infect Microbiol. 2022 Jun 23;12:888496. doi: 10.3389/fcimb.2022.888496 (PMC9260704; doi:10.3389/fcimb.2022.888496)
Supplement: Supplementary file 9 [file Table_4.docx]

| **Supplemental Table 4: Summary Statistics for Parasite Counts in 22 Tissues** | | | | | | |
| --- | --- | --- | --- | --- | --- | --- |
|  | **Pre-Controlled** | | | **Post-Controlled** | | |
| *Tissue* | *Mean* | *SD* | *N* | *Mean* | *SD* | *N* |
| Adrenal Gland | 105 | 112 | 6 | 0.67 | 0.58 | 3 |
| Aorta | 37.2 | 55.9 | 6 | 0.33 | 0.58 | 3 |
| Bone Marrow | 15.8 | 8.83 | 16 | 0.33 | 0.58 | 3 |
| Cerebellum | 3.83 | 4.59 | 6 | 0.00 | 0.00 | 4 |
| Cerebrum | 3.33 | 4.59 | 6 | 0.00 | 0.00 | 3 |
| Colon | 42.3 | 29.2 | 6 | 4.33 | 7.51 | 3 |
| Duodenum | 58.2 | 48.3 | 6 | 6.00 | 4.36 | 3 |
| Eye | 48.5 | 64.1 | 8 | 0.67 | 1.15 | 3 |
| Jejunum | 22.2 | 20.5 | 6 | 3.00 | 3.00 | 3 |
| Kidney | 93.2 | 62.5 | 6 | 1.00 | 0.00 | 4 |
| Liver | 47.2 | 26.3 | 6 | 0.33 | 0.58 | 3 |
| Lung | 86.3 | 42.7 | 6 | 3.00 | 2.94 | 3 |
| Mesenteric LN | 16.8 | 17.3 | 6 | 0.00 | 0.00 | 3 |
| Midbrain | 2.00 | 2.10 | 6 | 0.33 | 0.58 | 3 |
| Omentum | 12.0 | 8.88 | 6 | 0.33 | 0.58 | 3 |
| Skeletal muscle | 2.50 | 3.39 | 6 | 0.00 | 0.00 | 3 |
| Skin | 12.3 | 19.2 | 6 | 0.00 | 0.00 | 3 |
| Spleen | 171 | 141 | 6 | 0.33 | 0.57 | 3 |
| Stomach | 74.5 | 70.9 | 6 | 2.33 | 1.52 | 3 |
| Testis | 18.5 | 7.06 | 6 | 0.00 | 0.00 | 3 |
| Thymus | 14.3 | 12.6 | 6 | 0.0 | 0.0 | 3 |
| Ventricle | 49.0 | 27.0 | 6 | 1.67 | 2.89 | 3 |

**Supplemental Table 4. Summary statistics for parasite counts by tissue.** All tissue sections were blinded and examined by light microscopy under oil emersion at 1000x, and the number of parasites counted in 10 fields. Summary statistics are included here for the tissues of all animals for those necropsied prior to parasitemic control and after parasitemic control.
