# Supplementary material for: Plasmodium knowlesi Cytoadhesion Involves SICA Variant Proteins
Source: Front Cell Infect Microbiol. 2022 Jun 23;12:888496. doi: 10.3389/fcimb.2022.888496 (PMC9260704; doi:10.3389/fcimb.2022.888496)
Supplement: Supplementary file 10 [file Table_5.docx]

| **Supplemental Table 5: Hierarchical Linear Regression of Parasite Count vs. Day Post Infection, Parasitemia and Tissue** | | | | | | | | | | |  |  |  |  |  |
| --- | --- | --- | --- | --- | --- | --- | --- | --- | --- | --- | --- | --- | --- | --- | --- |
|  | **Model 1** | | | | | **Model 2** | | | | | **Model 3** | | | | |
|  | Est. | Std. Error | t value | P-value | Sig. | Est. | Std. Error | t value | P-value | Sig. | Est. | Std. Error | t value | P-value | Sig. |
| Intercept | 53.2 | 5.81 | 9.15 | < 2.00x10^-16^ | *** | 2.69 | 1.08 | 0.25 | 0.80 | NS | 4.41 | 9.26 | 0.48 | 0.63 | NS |
| DPI | 0.030 | 0.011 | 2.817 | 0.005 | ** | -0.06 | 0.26 | -0.23 | 0.82 | NS | -0.11 | 0.22 | -0.51 | 0.61 | NS |
| Parasitemia |  |  |  |  |  | 3.55x10^-4^ | 6.56x10^-5^ | 5.42 | 1.67x10^-7^ | *** | 3.60x10^-4^ | 5.61x10^-5^ | 6.41 | 1.13x10^-9^ | *** |
| Tissue |  |  |  |  |  |  |  |  |  |  |  |  |  |  |  |
| *Aorta* |  |  |  |  |  |  |  |  |  |  | 42.0 | 13.1 | 3.21 | 1.56x10^-3^ | ** |
| *Bone marrow* |  |  |  |  |  |  |  |  |  |  | -3.38 | 13.1 | -2.57 | 0.80 | NS |
| *Cerebellum* |  |  |  |  |  |  |  |  |  |  | -23.9 | 9.28 | -2.57 | 0.01 | * |
| *Cerebrum* |  |  |  |  |  |  |  |  |  |  | -25.6 | 13.1 | -1.96 | > 0.05 | NS |
| *Colon* |  |  |  |  |  |  |  |  |  |  | -26.0 | 13.1 | -1.99 | < 0.05 | * |
| *Duodenum* |  |  |  |  |  |  |  |  |  |  | 1.40 | 13.1 | 0.11 | 0.91 | NS |
| *Eye* |  |  |  |  |  |  |  |  |  |  | 12.5 | 13.1 | 0.96 | 0.34 | NS |
| *Jejunum* |  |  |  |  |  |  |  |  |  |  | 5.50 | 11.9 | 0.46 | 0.64 | NS |
| *Kidney* |  |  |  |  |  |  |  |  |  |  | -12.5 | 13.1 | -0.96 | 0.34 | NS |
| *Liver* |  |  |  |  |  |  |  |  |  |  | 31.0 | 12.3 | 2.49 | 0.01 | * |
| *Lung* |  |  |  |  |  |  |  |  |  |  | 3.29 | 13.1 | 0.25 | 0.801 | NS |
| *Lymph Node* |  |  |  |  |  |  |  |  |  |  | 27.7 | 12.4 | 2.22 | 0.03 | * |
| *Midbrain* |  |  |  |  |  |  |  |  |  |  | -17.0 | 13.1 | -1.30 | 0.19 | NS |
| *Muscle* |  |  |  |  |  |  |  |  |  |  | -26.8 | 13.1 | -2.05 | 0.04 | * |
| *Omentum* |  |  |  |  |  |  |  |  |  |  | -26.6 | 13.1 | -2.04 | 0.04 | * |
| *Skin* |  |  |  |  |  |  |  |  |  |  | -20.2 | 13.1 | -1.54 | 0.12 | NS |
| *Spleen* |  |  |  |  |  |  |  |  |  |  | -20.0 | 13.1 | -1.53 | 0.13 | NS |
| *Stomach* |  |  |  |  |  |  |  |  |  |  | 86.0 | 13.1 | 6.59 | 4.60x10^-10^ | *** |
| *Testis* |  |  |  |  |  |  |  |  |  |  | 22.2 | 13.1 | 1.70 | 0.09 | NS |
| *Thymus* |  |  |  |  |  |  |  |  |  |  | -15.6 | 13.1 | -1.19 | 0.23 | NS |
| *Ventricle* |  |  |  |  |  |  |  |  |  |  | -18.7 | 13.1 | -1.43 | 0.15 | NS |
|  | N =212; df = 210 Adjusted R^2^ = 0.12; F-statistic = 28.7;  P-value = 2.19x10^-7^; AIC = 2264 | | | | | N = 212; df = 209; Adjusted R^2^ = 0.22; F-statistic = 31.0;  P-value = 1.67x10^-12^; AIC = 2239 | | | | | N = 212; df = 188; Adjusted R2 = 0.43; F-statistic = 7.95  P-value = < 2.2x10^-16^; AIC = 2192 | | | | |
|  |  |  |  |  |  |  |  |  |  |  |  |  |  |  |  |

**Supplemental Table 5. Hierarchical Linear Regression of Parasite Count vs. Day Post Infection, Parasitemia and Tissue**.
Multiple linear regression model shows that tissue itself and circulating parasitemia are related to tissue count.
* p-value < 0.05; ** p-value < 0.005; *** p-value <0.0005; NS = not significant.
